# Exploring the association between home delivery and under-five mortality in Ethiopia: A bivariate multilevel mixed-effects analysis

**DOI:** 10.1371/journal.pone.0355621

**Published:** 2026-08-12

**Authors:** Wondaya Fenta Zewdia, Maru Zewdu Kassie, Melkamu A. Zeru, Asteray Assmie Ayenew

**Affiliations:** 1 Department of Statistics, College of Science, Bahir Dar University, Bahir Dar, Ethiopia; 2 Department of Statistics, College of Sciences, Assosa University, Assosa, Ethiopia; 3 Department of Epidemiology and Biostatistics, College of Medicine and Health Science, Bahir Dar University, Bahir Dar, Ethiopia; 4 Department of Midwifery, College of Medicine and Health Science, Bahir Dar University, Bahir Dar, Ethiopia; 5 Department of Obstetrics and Gynaecology, Monash University, Melbourne, Victoria, Australia; University of the Punjab, PAKISTAN

## Abstract

**Background of the study:**

Child mortality remains a major public health issue in Ethiopia, with under-five mortality rates among the highest in sub-Saharan Africa. Despite efforts to improve maternal and child health, many women still give birth at home without skilled assistance, which has been associated with adverse maternal and child health outcomes. Therefore, this study aimed to investigate the association and determinants of home delivery and under-five mortality (U5M) using a multilevel bivariate modeling approach.

**Methods:**

A cross-sectional study was conducted among 5,517 women with a birth history using data from the Ethiopian Mini Demographic and Health Survey (EMDHS) 2019. Data management and analysis were performed using STATA version 17 and R version 4.2.2. The chi-square test of association was used to examine the association between predictor and outcome variables, and multilevel bivariate analysis was conducted to identify significant factors associated with the outcome variables.

**Results:**

Out of 5,517 women with birth history, 2,842 (51.5%) were delivered at home, and 269 (4.87%) reported experiencing at least one under-five child death. As shown in the joint and marginal probabilities of home delivery and U5M, there was a significant association (χ² = 3.83, P-value < 0.0001). The adjusted odds ratio (AOR = 2.19, 95% CI: 1.89–2.62) indicates that mothers who delivered at home had 2.19 times higher odds of experiencing at least one under-five child death than mothers who delivered in a health facility.

**Conclusion:**

Overall, home delivery and U5M were significantly associated with a combination of individual, household, and community-level factors. Education, wealth, antenatal and postnatal care, and media exposure were associated with lower odds of both outcomes, while rural residence, too young or too old maternal age, and lower economic status were associated with higher odds**.** Regional disparities further indicate the need for targeted interventions. Expanding healthcare access in rural areas, improving female education, promoting antenatal and postnatal care, and increasing media-based health awareness campaigns may contribute to reducing the prevalence of home delivery and maternal experience of under-five child death in Ethiopia.

## Introduction

Child mortality remains a major global public health issue, with four million deaths occurring in the first month of life annually [1]**.** Under-five mortality (U5M), the risk of death between birth and age five, is a key indicator of societal well-being [2]. The U5M rate is a critical target of the Millennium Development Goals (MDGs) and Sustainable Development Goals (SDGs) [3]. The SDGs aim to reduce U5M to at least 25 deaths per 1,000 live births by 2030, emphasizing the importance of maternal health facilities in achieving this goal [4]. While the MDG 4 target of reducing child mortality by two-thirds was not fully met globally, certain regions, including East Asia and the Pacific, Latin America and the Caribbean, and 62 countries, made significant progress [5,6].

Home delivery, prevalent in developing countries, is significantly associated with U5M [7]. Despite improvements in health interventions, Sub-Saharan Africa continues to experience high under-five mortality rates [8]. Global health policies stress institutional deliveries as an important strategy for improving maternal and child health outcomes, but neonatal mortality decreases at a slower pace compared to child mortality, particularly in Sub-Saharan Africa [9,10]. According to the 2022 World Health Organization (WHO) report, Sub-Saharan Africa accounted for 57% of global under-five deaths (≈2.8 million; 95% CI: 2.5–3.3 million) despite representing only 30% of live births, and it recorded the world’s highest neonatal mortality rate (27 per 1,000 live births), followed by Central and Southern Asia (21 per 1,000) [11]. Similarly, a United Nation (UN) Inter-agency Group for Child Mortality Estimation analysis covering 2019 data found that the region contributed 55% of global U5M, with East Africa experiencing the highest infant mortality rate (92.2 per 1,000 live births) [12].

In Ethiopia, the prevalence of home deliveries declined from 73% in 2016 to 51% in 2019. However, a significant proportion of births continue to occur at home, which may be a key associated factor to the persistently high U5M rate in the country [13]. This pattern highlights an important public health concern, as home deliveries have been associated with limited access to skilled birth attendance, inadequate postnatal care, and adverse maternal and child health outcomes. Previous studies have identified multiple factors associated with childhood mortality, including socioeconomic conditions, maternal and child-related determinants, and healthcare utilization [14–18]. Based on the research [19], the most common causes of under-five mortality include acute respiratory infections, diarrhea, malaria, and birth complications, with neonatal disorders and infections remaining significant contributors in low- and middle-income countries (LMICs). Another study revealed that low parental education, younger maternal age, short birth intervals, non-immunized children, and inadequate toilet facilities were associated with higher under-five mortality, whereas access to safe healthcare was associated with lower under-five mortality [14].

The association between home deliveries and U5M remains a significant public health concern, as the place of delivery has been associated with neonatal and child survival outcomes [20–22]. Home births are often linked to higher mortality due to limited access to skilled care and emergency interventions [23,24]. Despite the decline in home deliveries in Ethiopia, they still account for a substantial proportion of births, and may contribute to the persistently high burden of U5M [25,26]. However, to date, few comprehensive studies have examined this relationship using a robust multilevel analytical approach in Ethiopia.

Our study addresses this gap by employing a bivariate multilevel analysis using data from the Ethiopian Mini Demographic and Health Survey (EMDHS) 2019. Unlike previous studies that used enumeration areas as the second-level unit in multilevel modeling, we adopt zones as a more policy-relevant and interpretable level of analysis. This approach accounts for the correlated nature of binary outcomes, enabling a more precise investigation of the association between home delivery and U5M. Given Ethiopia’s unique demographic profile, characterized by high birth and mortality rates, our findings may provide valuable evidence to support targeted maternal and child health interventions. Ultimately, our study seeks to inform strategies that enhance maternal and child health by improving understanding of the association between home delivery and maternal experience of under-five mortality in Ethiopia.

## Materials and methods

### Study area, data sources, and study population

This study was conducted in Ethiopia, the second most populous country in Africa, with over 110 million inhabitants as of 2022. Ethiopia is administratively divided into two chartered cities and nine regional states, which are further subdivided into zones, woredas, and kebeles. The study utilized cross-sectional, community-based data from the 2019 EMDHS, collected by the Ethiopian Public Health Institute (EPHI) in collaboration with the Federal Ministry of Health (FMoH) and the Central Statistical Agency (CSA), with technical assistance from the Demographic and Health Surveys (DHS) Program, ICF International, USA. A two-stage stratified cluster sampling method was employed. In the first stage, 305 enumeration areas were selected based on the 2019 Ethiopian Population and Housing Census (EPHC) frame, with 93 urban and 212 rural clusters sampled proportionally. In the second stage, 30 households were randomly selected from each cluster.

Data were extracted from the Kids Record (KR) files, which contain maternal and child health information, focusing on variables related to insufficient dietary diversity [27]. Ethiopia has conducted four full-scale Demographic and Health Surveys (DHS) in 2000, 2005, 2011, and 2016, along with two Mini DHS surveys in 2014 and 2019. The 2019 EMDHS, conducted between March 21 and June 28, 2019, serves as the data source for this study. We accessed the anonymized dataset from the DHS Program website https://dhsprogram.com/data/ on April 4, 2024. The data were de-identified before access, and the authors had no access to any information that could identify individual participants during or after data collection.

All women aged 15–49 years in each household were interviewed. The EMDHS 2019 survey covered 8,663 of the selected 8,794 households, yielding a response rate of 99%. A total of 8,885 women completed the interview out of 16,583 identified as eligible, resulting in a response rate of 99% [28]. Women who had at least one live birth were eligible for inclusion in the analysis. Consequently, 5,517 eligible woman-child paired observations were included after applying the necessary inclusion and exclusion criteria. Multiple imputation technique was used to handle missing data. Individual sampling weights (v005/1,000,000) were applied in all analyses to account for over- and under-sampling, where v005 is the sampling weight variable in the EMDHS 2019 dataset.

### Inclusion and exclusion criteria

**Fig 1:**
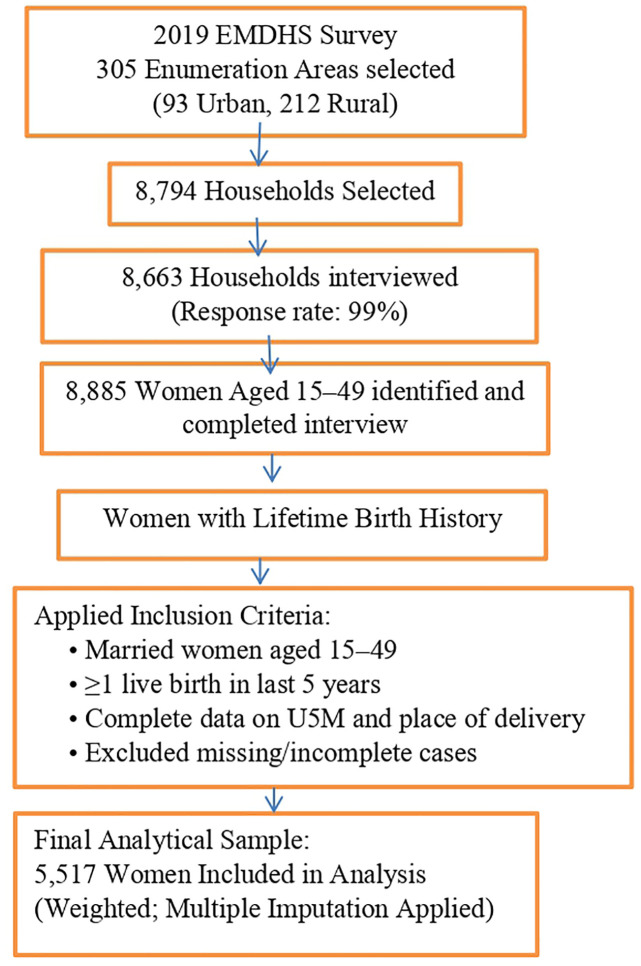
Flow chart showing the selection of study participants.

This study included women aged 15–49 years who were married and had at least one live birth within five years prior to the survey. Additionally, only children under five with complete data on under-five mortality (U5M) and place of delivery were considered. Participants with incomplete or missing information on key variables were excluded from the analysis. The flow chart illustrating the process of study participant selection is presented in Fig 1.

### Study variables

#### The response variables.

The two binary outcome variables in this study were maternal experience of under-five mortality (U5M) and place of delivery, as defined using the EMDHS-2019 dataset. U5M was operationalized as a mother-level binary variable, indicating whether a woman reported experiencing the death of at least one live-born child before reaching five years of age. Women who had experienced at least one such child deaths were coded as “1” (yes), and those who had not experienced any under-five child death were coded as “0” (no). Importantly, this measure does not represent the conventional under-five mortality rate (deaths per 1,000 live births). Rather, it reflects the proportion of mothers who experienced at least one under-five child death within their birth history. Accordingly, all reported percentages for U5M represent proportions of mothers, not child mortality rates.

Place of delivery was similarly defined as a binary maternal-level variable, categorized as home delivery (1) or health facility delivery (0), based on the most recent birth within five years preceding the survey.

#### Explanatory variables.

The study included a range of individual- and community-level explanatory variables for both outcome measures. This study considered predictors that were most strongly associated with the response variables: home delivery and U5M based on recent studies. These included:

- **Child-related factors**: Age (in months), sex, and twin status.
- **Maternal factors**: Current age, marital status, education level, contraceptive use, antenatal care follow-up, postnatal care, health worker counseling during pregnancy, and last birth by cesarean section.
- **Household factors**: Wealth index, household head’s sex, and access to mass media.
- **Community-level factors**: Region, place of residence, community education level, community wealth index, media exposure, and antenatal visitation. The list of variables with their measurements or definitions is briefly explained in Table 1.

**Table 1:** Description and measurement of predictor variables.

Level	Variable	Measurement/ Definition
Child-related factors	Age of child	Measured in completed months at the time of the survey.
	Sex of child	Coded as male or female.
	Twin status	Indicates whether the child was born as a single or multiple (twin) birth based on the mother’s birth history.
Maternal factors	Current age	Mother’s age in completed years, grouped as 15–18, 19–34, and 35–49 years.
	Marital status	Classified as married/living with partner or not currently married (widowed/divorced/separated/single).
	Educational level	Categorized as no formal education, primary, secondary, or higher, based on the highest level completed.
	Contraceptive use	Indicates whether the mother was using any modern contraceptive method at the time of the survey (Yes/No).
	Antenatal care (ANC) follow-up	Whether the mother received at least one ANC visit during her most recent pregnancy (Yes/No).
	Postnatal care (PNC)	Whether the mother or newborn received a postnatal check-up within two days after delivery (Yes/No).
	Last birth by cesarean section	Whether the most recent birth was delivered by cesarean section (Yes/No).
Household factors	Wealth index	Composite measure of household living standard derived from assets and housing materials using principal component analysis; categorized into quintiles (poor, middle, rich).
	Sex of household head	Coded as male or female, as reported by the respondent.
	Access to mass media	Indicates whether the household had exposure to radio, television, or newspapers at least once per week (Yes/No).
Community-level factors	Region	Administrative region or city administration of Ethiopia, as defined in the EMDHS 2019 dataset.
	Place of residence	Classified as urban or rural, based on the DHS sampling frame.
	Community education level	Proportion of women in each cluster with at least primary education; categorized as *low* or *high* using the national median value.
	Community wealth index	Average household wealth index at the cluster level; categorized as *low* or *high* based on the median.
	Community media exposure	Proportion of women in each cluster exposed to at least one type of mass media (radio, TV, or newspaper) at least once per week; categorized as *low* or *high*.
	Community antenatal visitation	Proportion of women in a cluster who attended at least one ANC visit during their most recent pregnancy; categorized as *low* or *high* based on the median.

### Statistical analysis

All analyses were conducted using the EMDHS 2019 dataset. Data management and descriptive analyses were performed in STATA (version 17), and the joint multilevel modeling was implemented in R (version 4.22) using maximum likelihood estimation.

### Rationale for joint multilevel modeling

The study examined two correlated binary maternal-level outcomes: home delivery (yes/no) and maternal experience of under-five child death (yes/no). Because these outcomes may occur together within the same woman, fitting two separate logistic regression models would ignore their correlation. Therefore, a bivariate multilevel mixed-effects logistic regression model was used to jointly analyze both outcomes while accounting for clustering of women within communities (Zones).

The data had a two-level structure, with women at Level 1 and communities at Level 2. A random intercept was specified at the community level to capture unobserved contextual influences, allowing women within the same community to be more similar to each other than to women from different communities. The variance of this random intercept represents the degree of between-community heterogeneity.

### Bivariate logistic multilevel model

The bivariate multilevel logistic regression model simultaneously estimates one logistic regression equation for each outcome while allowing the two outcomes to be statistically correlated through an association parameter. This approach improves estimation efficiency by accounting for the dependence between the two binary outcomes while adjusting for both individual- and community-level characteristics [28–32]. The Multilevel binary having a two-level model is given by;


$$\begin{array}{c} ogit\left(\pi_{ij}\right)=log\left(\frac{\pi_{ij}}{\text{1}-\pi_{ij}}\right)=\gamma_{\text{0}\text{0}}+\gamma_{k\text{0}}X_{kij}+\gamma_{\text{0}q}Z_{qj}+U_{\text{0}j}+U_{kj}U_{kj}\ldots \end{array}$$ (1)

Where $\gamma_{\text{0}\text{0}}$ is the average regression intercept, $X_{kij}$ represents the 𝑘 level-one covariates, and $Z_{qj}\;$represents the 𝑞 level-two covariates, whereas $\gamma_{k\text{0}}$ and $\gamma_{\text{0}q}$ are the corresponding regression coefficients for level-one and level-two explanatory variables; $U_{\text{0}j}\;$and $U_{kj}\;$are the random effect of the model parameters at level two. It is expected that the vector of random effects $U_{\text{0}j},U_{\text{1}j},U_{\text{2}j},\ldots ,\ldots ,\ldots ,U_{kj}$ follow a normal distribution with mean zero and variance$\;\sigma_{u\text{0}}^{\text{2}}$. Akaike’s information criterion (AIC) and the Bayesian information criterion (BIC) determine the best multilevel model out of the multilevel models.

The intra-class correlation (ICC) was used to quantify the degree of similarity between individuals within the same neighborhood. The ICC value ranges from 0 to 1, where higher values indicate greater within-cluster similarity.

The ICC is given by:


$$\begin{array}{c} ICC=\rho \left(Y_{ij},Y_{i\text{'}j}\right)=\frac{cov\left(Y_{ij},Y_{i\text{'}j}\right)}{var\left(U_{\text{0}j}\right)+var\left(\varepsilon_{ij}\right)}=\frac{\sigma_{u\text{0}}^{\text{2}}}{\sigma_{u\text{0}}^{\text{2}}+\sigma_{e}^{\text{2}}}\;=\;\frac{\sigma_{u\text{0}}^{\text{2}}}{\sigma_{u\text{0}}^{\text{2}}+\frac{\pi^{\text{2}}}{\text{3}}} \end{array}$$ (2)

Where, $\sigma_{e}^{\text{2}}$ is the variance of individual-level units and $\sigma_{u\text{0}}^{\text{2}}$ is the variance of the higher-level residual errors. In the multilevel logit model, the estimated level-1 variance is the standard logistic distribution, $\frac{{\pi \;}^{\text{2}}}{\text{3}}$, which is considered as level-1 residual variance${(\sigma }_{e}^{\text{2}}),\;i.e\;\sigma_{e}^{\text{2}}=\;\frac{{\pi \;}^{\text{2}}}{\text{3}}\;\approx \;\text{3}.\text{29}$. In the absence of a multilevel structure, a single-level individual analysis is appropriate. Proportional change in variance (PCV) was calculated to assess the reduction in between-community variance after inclusion of covariates.

Let $Y_{\text{1}}$ and $Y_{\text{2}}$ represent home delivery and U5M proportion, respectively. The two bivariate binary responses can be denoted as a vector Y =$\;(\gamma_{\text{1}\text{1}}\;\gamma_{\text{0}\text{1}}\;\gamma_{\text{1}\text{0}}\;\gamma_{\text{0}\text{0}}{]}^{T}$, with the joint probabilities structured as shown in Table 2.

**Table 2:** Joint probability of the response variables.

U5M(Y_2_)			
Home Delivery $({𝐘}_{1})$	1	0	Total
1	$\gamma_{\text{11}}$	$\gamma_{\text{10}}$	$\gamma_{\text{1}}$
0	$\gamma_{\text{01}}$	$\gamma_{\text{00}}$	${\text{1}\;-\;\gamma }_{\text{1}}$
Total	$\gamma_{\text{2}}$	${\text{1}\;-\;\gamma }_{\text{2}}$	1

The association parameter (Ψ) quantifies the strength and direction of the relationship between home delivery and maternal experience of under-five child death after accounting for the explanatory variables and community-level random effects. After exponentiation, exp(Ψ) represents the adjusted odds ratio describing this association. Values greater than one indicate that mothers who delivered at home had higher odds of experiencing at least one under-five child death than mothers who delivered in a health facility, whereas values less than one indicate lower odds, after adjustment for the covariates included in the model. Thus, the association parameter summarizes the adjusted relationship between the two outcomes rather than implying a causal effect of place of delivery on under-five child death. Parameters were estimated using maximum likelihood via the Newton-Raphson iterative algorithm. Model estimates were presented as adjusted odds ratios (AORs) with 95% confidence intervals, and statistical significance was assessed using a two-sided p-value <0.05. [30,33].

### Ethics approval and consent to participate

All methods were conducted following the guidelines of the Demographic and Health Survey (DHS) program. Informed consent was waived by the International Review Board of the DHS program data archivists. Upon submission of the consent paper to the DHS Program, a letter of permission to download the dataset for this study was obtained. The data were solely used for this study and could not be shared with other researchers without DHS consent. The methods adhered to the ethical standards of the Declaration of Helsinki.

## Results

The distribution of various independent variables (covariates) and their association with home delivery and U5M in Ethiopia are presented in Table 3. The chi-square (χ²) p-values indicate the statistical significance of the association between each independent variable and the two outcome variables (home delivery and U5M). Of the 5,517 women with a birth history, 2,842 (51.5%) delivered at home, and 269 (4.87%) experienced at least one U5M.

**Table 3:** Distribution and Association of covariate with Home Delivery and U5M.

Independent Variables with Categories	Frequency (%)	Home Delivery		U5M	
		Yes (%)	$\chi^{2}-\;$P value	Yes(%)	$\chi^{2}-\;$P value
**Age of Mother (in Year)**					
15-18 19-34 35-49	176(3.2)	82(2.9)	0.0001	14(5.2)	0.0000
	4065(73.7)	2026(71.3)		179(66.5)	
	1146(23.1)	734(25.8)		76(28.3)	
**Religion of Mother**					
Orthodox Catholic Protestant Muslim Others	1857(33.7)	764(26.9)	0.0001	76(28.3)	< 0.0001
	17(0.3)	17(0.6)		0(0.0)	
	1458(26.4)	847(29.8)		48(17.8)	
	2095(38)	1142(40.2)		140(52.0)	
	90(1.6)	72(2.5)		5(1.9)	
**Place of residence**					
Urban Rural	1362(24.7)	399(14.0)	0.0001	44(16.4)	< 0.0001
	4156(75.3)	2443(86.0)		225(83.6)	
**Mother Educational Level**					
No Education Primary Secondary Higher	2960(58.5)	1955(68.8)	0.0000	158(58.5)	0.0001
	1949(35.3)	810(28.5)		94(34.8)	
	415(7.5)	64(2.3)		14(5.2)	
	194(3.5)	13(0.7)		4(1.5)	
**Region**					
Tigray Afar Amhara Oromia Somali Benishangul SNNPR Gambela Harari Addis Ababa Dire Dewa	371(6.7)	98(3.4)	0.0001	14(5.2)	<0.0001
	86(1.6)	62(2.2)		5(1.8)	
	1047(19.0)	466(16.4)		44(16.2)	
	2206(40.0)	1297(45.0)		113(41.7)	
	408(7.4)	310(8.9)		41(15.1)	
	67(1.2)	21(0.7)		5(1.8)	
	1103(20.0)	576(20.3)		41(15.1)	
	24(0.4)	7(0.2)		2(0.7)	
	17(0.3)	6(0.2)		1(0.4)	
	156(2.8)	7(0.2)		3(1.1)	
	30(0.5)	9(0.3)		2(0.7)	
**Wealth Index**					
Poor Middle Rich	2518(45.6)	1769(62.2)	0.0001	150(55.8)	< 0.0001
	1041(18.9)	577(20.3)		47(17.5)	
	1958(35.5)	496(17.5)		72(26.8)	
**Birth Interval of Child**					
High Low	1105(20)	733(25.8)	0.0001	96(35.7)	< 0.0001
	4413(80)	2109(74.2)		173(64.3)	
**No of Children Under Five in the House**					
High(>3)	4778(86.6)	2300(80.2)	0.0001	254(94.4)	0.0001
Low(<=3)	739(13.4)	543(19.8)		15(5.6)	
**Sex of House Hold Head**					
Male Female	4331(78.5)	2191(77.1)	0.000	219(81.4)	0.0001
	1188(21.5)	652(22.9)		50(18.6)	
**Media Exposures**					
Yes No	1882(34.1)	613(21.6)	0.0001	78(29.0)	0.0001
	3635(65.9)	2229(78.4)		191(71.0)	
**During Pregnancy Counseling Health Worker**					
Yes No	2074(37.6)	620(21.8)	0.0001	60(22.4)	0.0000
	3443(62.4)	2222(78.2)		209(77.6)	
**Current Contraceptive Use**					
Yes No	2242(40.6)	901(31.7)	0.0001	72(26.4)	0.0000
	3275(59.4)	1941(68.3)		197(73.6)	
**Postnatal Check within 2 Months**					
Yes No	535(9.7)	147(5.2)	0.0000	13(4.8)	0.000
	4983(90.3)	2695(94.8)		257(95.2)	
**Number of Anti Natal Care (ANC) visit**					
aw Visit No Visit	1009(18.3)	863(30.8)	0.0001	65(24.3)	0.0004
	4509(50)	1979(69.2)		204(75.8)	
**Marital Status**					
Married	5184(94.0)	2669(93.9)	0.0000	250(92.9)	0.0000
Not	333(6.0)	173(6.1)		19(7.2)	
**Community level Anti Natal Visit**					
Low	3054(55.4)	2046(72.0)	0.0000	177(65.4)	0.0000
High	2463(44.6)	796(28.0)		92(34.6)	
**Community level Wealth Index**					
Low	2712(49.2)	1677(59.0)		154(57.0)	0.0000
High	2805(50.8)	1165(41.0)		116(43.0)	
**Community level Education**					
Low	3394(61.5)	1929(67.9)	0.0000	187(69.5)	0.0000
High	2124(38.5)	913(32.1)		82(30.5)	
**Community level Media Exposure**					
Low	2382(43.2)	1440(50.7)	0.0000	146(54.3)	0.0000
High	3135(56.8)	1402(49.3)		123(45.7)	

Younger mothers (15–18 years) had a lower proportion of home deliveries compared with older mothers, while the highest proportion of mothers reporting at least one under-five child death was observed among women aged 35–49 years. The association between maternal age and both home delivery and U5M was statistically significant (p-value < 0.0001). Religion also influenced home delivery and U5M, with Muslim mothers showing the highest proportion of both outcomes. Place of residence was another important factor. Rural mothers had a much higher proportion of home deliveries (86%) compared to urban mothers (772; 14%). Similarly, U5M was higher among rural residents (4,612; 83.6%). As indicated by the chi-square test (p-value < 0.0001), place of residence was strongly associated with both outcomes.

Regarding education, women with no formal education had the highest proportion of home deliveries (68.8%), and the proportion of mothers reporting at least one under-five child death was also highest among women with no formal education. Higher levels of education were significantly associated with lower proportions of both home delivery and U5M (p < 0.0001). The wealth index also influenced both outcomes, with poorer households showing a higher prevalence of home delivery (62.2%) and U5M (55.8%). This association was statistically significant (p-value < 0.0001).

Sex of the household head also played a role. Male-headed households had slightly higher proportions of home delivery (77.1%) and U5M (81.4%), and this association was statistically significant (p < 0.0001). Regarding media exposure, mothers with media exposure had significantly lower proportions of home delivery (21.6%) and U5M (29%), whereas those without media exposure had higher proportions of home delivery (78.4%) and U5M (71%). Media exposure was significantly associated with both outcomes (p < 0.0001).

Healthcare access and utilization were crucial factors for both outcomes. Women who received antenatal care (ANC), counseling from health workers, and postnatal check-ups within two months had lower proportions of home delivery and maternal experience of under-five child death. For example, pregnancy counseling was associated with reduced proportions of home delivery (21.8%) and U5M (22.4%), whereas lack of counseling increased the likelihood of both outcomes. Similarly, non-use of contraceptives was associated with higher proportions of home delivery and U5M.

Regarding community-level factors, low community ANC coverage, low community wealth index, low community education level, and low community media exposure were significantly associated with higher proportions of home delivery and U5M (p < 0.0001).

**Fig 2:**
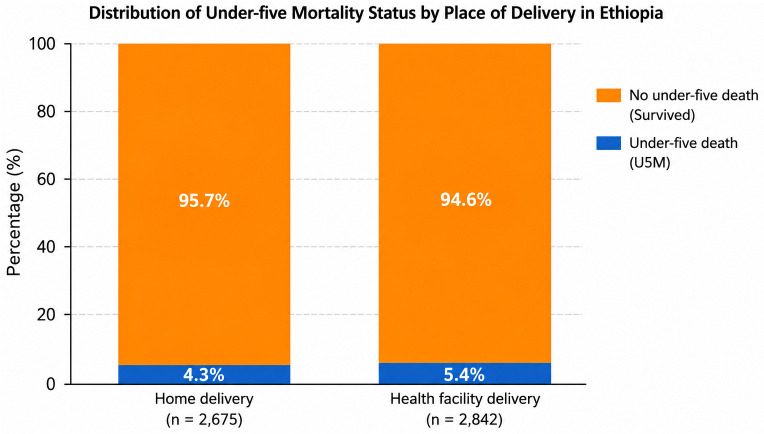
Distribution of under-five mortality status by place of delivery in Ethiopia. **Figure description:** The figure illustrates the distribution of maternal experience of under-five mortality by place of delivery in Ethiopia. Percentages represent the proportion of women who did and did not experience at least one under-five child death within each place of delivery category.

Fig 2 shows the distribution of maternal experience of under-five mortality by place of delivery. Among the 2,675 women who delivered at home**,** 115 (4.3%) experienced at least one under-five child death, while 2,560 (95.7%) did not experience under-five child death. Similarly, among the 2,842 women who delivered in a health facility**,** 154 (5.4%) experienced at least one under-five child death, whereas 2,688 (94.6%) did not. These descriptive findings indicate that the proportion of women who experienced under-five child death was slightly higher among those who delivered in health facilities than among those who delivered at home. However, these crude percentages do not account for differences in maternal, household, or community characteristics and therefore should not be interpreted as evidence of a causal relationship between place of delivery and under-five mortality. The independent association between place of delivery and under-five mortality was subsequently assessed using the multilevel bivariate logistic regression model.

### Joint and marginal probability of home delivery and U5M

The joint and marginal probabilities of home delivery and U5M, along with their association and odds ratio is presented in Table 4. The chi-square test (χ² = 14.83, P-value < 0.0001) indicates a statistically significant relationship between home delivery and U5M, indicating a statistically significant association between place of delivery and maternal experience of under-five child death. Regarding the odds ratio, mothers who delivered at home had 2.19 times higher odds of reporting at least one under-five child death than mothers who delivered in a health facility (OR = 2.19, 95% CI: 1.89–2.62). Since the confidence interval (CI) does not include 1, the association is statistically significant. This finding indicates a statistically significant association between home delivery and maternal experience of under-five child death. However, because of the cross-sectional design, the observed association should not be interpreted as evidence that place of delivery causes or prevents under-five child death.

**Table 4:** Joint and marginal probability of Home Delivery and U5M.

Responses		U5M		Marginal of Home Delivery	Odds Ratio (95% CI)
		No	Yes		
**Home Delivery**	**No**	2688(51.2)	154(57.2)	2842(51.5)	2.19(1.89, 2.62)
	**Yes**	2560(48.8)	**115(42.8)**	2675(48.5)	
**Marginal of U5M**		3139(95.2)	269(4.8)	5517(1)	

**Measure of Association,** $\;\chi^{2}\;=14.83,\;$ P_ value=0.0001**.**

### Model comparison

Table 5 presents the model comparison results for four different models (Model I to Model IV) based on variance, ICC, PCV, and model selection criteria (AIC and BIC) for both outcome variables at a time. The Pearson chi-square/DF values are also provided to assess the goodness of fit. In conclusion, among the four models, Model IV appears to be the best fit, as it has the lowest variance, the highest PCV (explaining 54% of the variance), and the smallest AIC and BIC values. The ICC value of 0.21 indicates that approximately 21% of the total variation was attributable to differences between communities (Zones), supporting the use of a multilevel modeling approach. The Pearson chi-square/DF values indicate that all models fit well, with Model II showing an exact value of 1. However, Model IV is preferred overall due to its superior explanatory power and improved model fit together for both outcomes at the same time. The final bivariate multilevel model jointly estimated both binary outcomes while accounting for their correlation and community-level clustering.

**Table 5:** Model Comparison for report among MI, MII, MIII and MIV and Variability between household levels.

Parameter	Model-I	Model-II	Model-III	Model-IV
Variance	0.8967	0.8658	0.7791	0.4150
ICC	Ref			0.21
PCV(R^2^)	Ref			0.54
AIC (smaller is better)	12381.06	7541.53	7524.07	7503.45
BIC (smaller is better)	12385.39	7681.86	7694.62	7591.28
Pearson Chi-Square/DF	0.94	1.00	0.91	0.94

### Parameter estimates of the multilevel bivariate modeling of home delivery and U5M

The results of a multilevel bivariate model analyzing the association between various socio-demographic and household-level factors with home delivery and U5M are presented in Table 6. The table includes estimated coefficients (Estimate), adjusted odds ratios (AOR), and 95% confidence intervals (CI) for the estimate of each factor.

**Table 6:** Parameter estimates of the multilevel bivariate modeling of Home Delivery and U5M.

Variables	Home Delivery		U5M	
	Estimate (95% CI)	AOR	Estimate (95% CI)	AOR
**Intercept**	-0.4113(-0.425, 0.9584)	0.662	-0.4113(-0.425, 0.9584)	0.662
**Mother’s Age in year**				
15-18 19-34 35-49	0.53(0.15, 0.91)**	1.699	0.73(0.09, 1.37)**	2.075
	Ref	1	Ref	1
	0.17(0.01,0.34)*	1.185	0.30(0.03, 0.61)**	1.350
**Religion Respondents**				
Orthodox Catholic Protestant Muslim Others	0.53(0.16, 1.20)**	1.699	0.23(0.32, 1.47)*	1.258
	-2.91(--6.41, 0.59)	0.054	-1.85(-7.32, 3.62)	0.157
	0.07(-0.59, 0.72)	1.072	-0.03(-1.27, 1.22)	0.970
	1.06(0.39, 1.72)**	2.886	-0.03(-1.27, 1.22)	0.970
	Ref	1	Ref	1
**Place of Residence**				
Urban Rural	Ref	1	Ref	1
	0.46(0.24, 0.69)***	1.584	0.72(0.28, 1.17)***	2.054
**Mother Educational Level**				
No education	0.87(0.26, 1.48)***	2.386	0.25(0.17, 0.77)**	1.284
Primary	Ref	1	Ref	1
Secondary	-0.59(-0.92, -0.26)**	0.554	-0.13(-0.74, 0.49)	0.878
Higher	-0.81(-0.97, -0.65)**	0.445	-0.68(-1.85, -0.49)**	0.506
**Region**				
Tigray Afar Amhara Oromia Somalia Benishangul SNNPR Gambela Harari Addis Ababa Dire Dewa	3.11(0.97, 5.25)***	22.42	-0.16(-1.75, 1.43)	0.852
	1.41(-0.81, 3.64)	4.096	0.17(-1.77, 2.10)	1.185
	2.37(0.24, 4.49)**	10.697	0.11(-1.39, 1.61)	1.116
	2.25(0.15, 4.34)**	9.488	-0.07(-1.47, 1.34)	0.932
	1.84(-0.31, 3.98)***	6.296	2.11(0.53, 3.69)***	8.248
	3.14(0.90, 5.38)**	23.103	1.05(-0.88, 2.97)	2.857
	2.74(0.63, 4.84)**	15.487	0.05(-1.44, 1.52	1.051
	3.32(0.90 5.74)**	27.660	0.77(-1.64, 3.19)	2.159
	1.93(-0.63, 4.49)	6.889	0.49(-2.51, 3.48)	1.632
	Ref	1	Ref	1
	2.24(0.19, 4.65)***	9.393	0.81(-1.61, 3.23)**	2.247
**Wealth Index of Household**				
Poor Middle Rich	0.32(0.13, 0.51)**	1.377	0.17(0.12, 0.55)**	1.185
	Ref	1	Ref	1
	-1.27(-1.48, -1.06)*	0.281	-0.25(-0.67, -0.17)*	0.779
**Number of Under-five in Household**				
Low(<=3) High(>3)	Ref	1	Ref	1
	-0.54(-0.77, 0.33)	0.583	-1.82(-2.49, 1.23)	0.162
**Birth Interval of Child**				
Low High	Ref	1	Ref	1
	-0.06(-0.37, 0.25)	0.942	0.22(-0.44, 0.88)	1.246
**Sex of House Hold Head**				
Male Female	Ref	1	Ref	1
	0.02(-0.09, 0.53)	1.020	0.22(-0.19,0.42)	1.246
**Pregnancy Counseling**				
No	Ref	1	Ref	1
Yes	1.408(-0.654, 1.67)	4.088	-0.546(-0.145, 0.856)	0.579
**Media Exposures**				
No Yes	Ref	1	Ref	1
	-0.09(-0.28, -0.03)*	0.913	-0.07(-0.43, -0.27)*	0.932
**Marital Status**				
Not married	Ref	Ref	1	Ref
Married	0.18(-0.11, 0.48)	1.197	-0.07(-0.64, 0.49)	0.932
**Current Contraceptive Use**				
No Yes	Ref	1	Ref	1
	-0.24(-0.40, -0.09)**	0.786	-0.19(-0.52, -0.13)**	0.827
**Postnatal Check within 2 months**				
No Yes	Ref	1	Ref	1
	-0.48(-0.73, -0.23)**	0.619	-0.47(-1.08, -0.14)**	0.625
**Number ANC Visit**				
No Visit Visit	Ref	1	Ref	1
	-1.23(-1.45, -1.01)***	0.292	-0.24(-0.11, 0.58)	0.787
**Community Level Anti Natal Visit**				
Low High	0.71(0.34, 1.08)**	2.034	0.45(0.22, 1.17)**	1.568
	Ref	1	Ref	1
**Community Level Wealth Index**				
Low High	0.15(0.02, 0.58)**	1.162	0.69(0.06, 1.39)**	1.993
	Ref	1	Ref	
**Community Level Education**				
Low High	0.14(0.02, 0.49)**	1.150	0.12(0.05, 0.72)**	1.127
	Ref	1	Ref	1
**Community Level Media Exposure**				
Low	0.18(0.03, 4.31)**	1.197	0.08(0.06, 0.81)**	1.083
High	Ref	1	Ref	1

Measure of dependency; [OR (95% CI) =3.501 (1.267, 6.713)] ***.

* = significant at 𝑎 = 0.05, ** = significant at 𝑎 = 0.01 and *** = significant at 𝑎 = 0.001.

Most importantly, for the bivariate multilevel model, the overall AOR is greater than one [AOR (95% CI) = 3.501 (1.267, 6.713)], indicating a statistically significant positive association between home delivery and maternal experience of under-five child death after adjusting for individual- and community-level covariates. In other words, mothers who delivered at home had higher odds of reporting at least one under-five child death than mothers who delivered in a health facility. However, because of the cross-sectional design, this association should not be interpreted as evidence of a causal relationship. Maternal age was significantly associated with both home delivery and maternal experience of under-five child death. Mothers aged 15–18 had 1.699 times higher odds of delivering at home and 2.075 times higher odds of U5M compared to the reference group (19–34 years). Additionally, mothers aged 35–49 had slightly elevated odds of home delivery (1.185) and U5M (1.350), though weaker compared to the youngest group.

Place of residence was significantly associated with both outcomes. Being too young or too old as a mother exacerbates the problems of home delivery and U5M. Mothers who follow the Orthodox religion have a higher association with both home delivery and U5M compared to those of other religions in Ethiopia. Similarly, mothers who follow Islam also face higher rates of home delivery. Rural-dwelling mothers had 1.584 times higher odds of home delivery and 2.054 times higher odds of U5M compared to urban mothers These findings indicate that rural residence was associated with higher odds of both outcomes.

Maternal education was significantly associated with both outcomes. Women with no formal education had 2.386 times higher odds of home delivery and 1.284 times higher odds of U5M compared to those with primary education. Higher education significantly reduced the odds of both home delivery (AOR = 0.445) and U5M (AOR = 0.506), emphasizing the importance of female education in improving maternal and child health outcomes. Importantly, mothers with secondary education were less likely associated with home delivery compared to those with only primary education. Additionally, mothers who had experience using contraceptives were less likely associated with home delivery and U5M compared to non-users.

Regional disparities were also notable. The highest odds of home delivery were observed in Gambela (AOR = 27.66), Benishangul (AOR = 23.10), and Tigray (AOR = 22.42), with similar trends in Amhara, Oromia, and SNNPR regions. Regarding child mortality, Somalia (AOR = 8.248) and Dire Dawa (AOR = 2.247) exhibited the highest odds. These regional estimates should be interpreted cautiously because some regions had relatively small sample sizes, which may have contributed to unstable estimates and the large adjusted odds ratios observed. Wealth index was another important factor. Poor households had 1.377 times higher odds of home delivery and 1.185 times higher odds of U5M compared to middle-income households. Conversely, wealthier households had a lower proportion of home delivery (AOR = 0.281) and U5M (AOR = 0.779), reinforcing the association between economic status and child health outcomes. Media exposure was also associated with lower odds of both home delivery (AOR = 0.913) and maternal experience of under-five child death (AOR = 0.932). Likewise, postnatal care attendance within two months was associated with lower odds of home delivery (AOR = 0.619) and maternal experience of under-five child death (AOR = 0.625).

Antenatal care (ANC) visits were another strongly important factor. Mothers with no ANC visits had significantly lower odds of home delivery (AOR = 0.292), highlighting the importance of prenatal checkups. Regarding community-level factors, higher community ANC coverage, community wealth, community education, and community media exposure were all associated with lower odds of home delivery and maternal experience of under-five child death. These findings suggest that favorable community characteristics were associated with lower odds of both outcomes in Ethiopia.

## Discussion

This study examined the determinants of home delivery and U5M using a multilevel bivariate modeling approach. The findings highlight the significant influence of maternal, household, and community-level factors on both outcomes, emphasizing the need for targeted interventions to improve maternal and child health. The results revealed that, out of 5,517 reproductive-aged women who had been married and had at least one child born within the five years prior to the survey, 2,842 (51.5%) were delivered at home, and 269 (4.87%) experienced at least one U5M.

The study revealed that home delivery was positively associated with U5M. This finding aligns with previous studies [20,34–36], which also reported an association between place of delivery and child survival outcomes**.** It also aligns with the findings of [22], which reported that health facility delivery was associated with lower neonatal mortality in low- and middle-income countries. As shown in the analysis of home delivery and its determinants, 51.5% of births occurred at home, with rural residence, lower maternal education, and economic disadvantage being major associated factors. Women in rural areas had significantly higher odds of delivering at home compared to their urban counterparts, which may reflect differences in healthcare access, infrastructure, and cultural practices. This finding aligns with previous studies in Ethiopia [23,37–39] and other low-income countries [40,41], where distance to healthcare facilities and transportation challenges have been reported to be associated with institutional delivery.

Maternal education was significantly associated with both home delivery and maternal experience of under-five child death, with higher educational attainment being associated with lower odds of both outcomes**.** Educated women are more likely to seek healthcare services, be aware of birth-related risks, and have greater decision-making autonomy. This finding aligns with previous studies [37,39,42,43], which reported that home delivery and under-five mortality (U5M) rates were lower among educated mothers compared to uneducated mothers. Similarly, wealth status played a crucial role, as poorer households had higher odds of home delivery and U5M compared to wealthier ones, reinforcing the financial burden of facility-based deliveries. These findings underscore the importance of expanding maternal education programs and addressing economic barriers to ensure safe deliveries. This association is well-documented in the literature, where lower wealth indices correlate with increased under-five mortality rates [23,39,43,44]. In their findings, economic constraints often limit access to quality healthcare and nutrition, contributing to higher mortality.

Utilization of ANC services and postnatal check-ups was associated with lower odds of home delivery and U5M. Mothers attending four or more ANC visits were more likely to deliver in health facilities and less likely to report experiencing under-five child death**.** This finding is supported by research indicating that adequate ANC visits are associated with reduced under-five mortality [45,46]. In their findings, antenatal care visit decreases the likelihood of under-five mortality. Additionally, our study identifies significant regional disparities in home delivery practices and U5M rates across Ethiopia. Regions such as Afar, Gambela, Somali, and Benishangul-Gumuz exhibit higher odds of home delivery and U5M compared to Addis Ababa. These variations may reflect differences in healthcare infrastructure, cultural practices, and socioeconomic conditions. However, the relatively large regional odds ratios should be interpreted cautiously because they may partly reflect small sample sizes or residual heterogeneity across regions. Similar regional disparities have been reported in other studies, underscoring the need for region-specific interventions [23,39].

The insights from this study underscore the necessity for targeted interventions addressing factors associated with home delivery and under-five mortality in Ethiopia. Strategies should focus on enhancing maternal education, improving economic conditions, and increasing access to quality antenatal and postnatal care, particularly in rural and underserved regions. Although the cross-sectional design does not permit causal inference, the observed associations provide useful evidence for strengthening maternal and child health programs. In conclusion, our research highlights the multifaceted factors associated with home delivery practices and under-five mortality in Ethiopia. By aligning our findings with existing literature, we identify important areas for strengthening maternal and child health services in the country.

## Conclusion

This study identified important factors associated with home delivery and maternal experience of under-five child death in Ethiopia. Maternal age, place of residence, education, household wealth, ANC visits, and regional disparities were significantly associated with both outcomes. Women who were older, lived in rural areas, or belonged to poorer households had higher odds of reporting at least one under-five child death, whereas higher maternal education, ANC attendance, and urban residence were associated with lower odds. Women who delivered at home also had higher odds of reporting at least one under-five child death than women who delivered in health facilities.

Because this study was based on cross-sectional survey data, these findings should be interpreted as statistical associations rather than causal effects. Regional disparities further emphasize the need for targeted, location-specific interventions. Strengthening maternal health services, expanding education, improving economic conditions, and promoting ANC utilization are important strategies for improving maternal and child health in Ethiopia. The observed associations may help policymakers identify populations that could benefit from targeted maternal and child health interventions. This study has important public health implications, particularly for strengthening the integration of maternal and child health services with community education and health promotion. The findings support continued efforts to improve access to quality maternal healthcare, increase awareness of facility-based delivery services, and strengthen community-based maternal and child health programs. Further longitudinal research is recommended to better understand the temporal relationship between home delivery and under-five child death and to explore potential causal pathways.

### Strength and limitations

This study has strengths and limitations. One of its key strengths is the use of a large, nationally representative dataset, enhancing the generalizability of the findings to the Ethiopian population. Additionally, the application of multilevel modeling allows for a more comprehensive analysis by accounting for both individual- and household-level variations. The study also provides critical insights into the regional disparities in home delivery and U5M, highlighting the need for targeted interventions.

However, certain limitations should be acknowledged. First, because this study was based on a cross-sectional survey, it identifies statistical associations but cannot establish temporal or causal relationships between home delivery and maternal experience of under-five child death. The reliance on self-reported data may introduce recall bias. Additionally, some potential predictor variables, such as the child’s birth weight, clinical variables, and maternal health conditions like diabetes mellitus and hypertension, were not collected. Moreover, we only considered data from 64 Ethiopian administrative zones, as data from 8 zones were not included in the 2019 EMDHS data collection. Despite these limitations, the study provides valuable evidence to inform maternal and child health policies, emphasizing the need for region-specific strategies to strengthen maternal and child health services in Ethiopia. Therefore, we recommend that future studies on maternal and child health be conducted by incorporating data from all Ethiopian zones and including potential variables that could not be included in this study, once the EDHS releases data encompassing these factors. Prospective or longitudinal studies would also be valuable for investigating the temporal relationship between home delivery and maternal experience of under-five child death.
