# Supplementary material for: Exploring the association between home delivery and under-five mortality in Ethiopia: A bivariate multilevel mixed-effects analysis
Source: PLoS One. 2026 Aug 12;21(8):e0355621. doi: 10.1371/journal.pone.0355621 (PMC13465812; doi:10.1371/journal.pone.0355621)
Supplement: Supplementary file 1 [file pone.0355621.s001.pdf]

PLOS ONE

Exploring the association between home delivery and under-five mortality in Ethiopia: A bivariate multilevel mixed-effects analysis

--Manuscript Draft--

**Manuscript Number:**	PONE-D-25-42341
**Article Type:**	Research Article
**Full Title:**	Exploring the association between home delivery and under-five mortality in Ethiopia: A bivariate multilevel mixed-effects analysis
**Short Title:**	Home Delivery and Under-Five Mortality in Ethiopia
**Corresponding Author:**	Maru Zewdu Kassie, MSc in Biostatistics<br>Assosa University<br>Assosa, BGRS ETHIOPIA
**Keywords:**	Home delivery, Under-five mortality, bivariate multilevel analysis, Women, Health Facility Births
**Abstract:**	Background of the study: Child mortality remains a major public health issue in Ethiopia, with under-five mortality rates among the highest in sub-Saharan Africa. Despite efforts to improve maternal and child health, many women still give birth at home without skilled assistance, increasing the risk of neonatal and child mortality due to childbirth complications, inadequate postnatal care, and infections. So, this study aimed to investigate the association and determinants of home delivery and under-five mortality (U5M) using a multilevel bivariate modeling approach.<br>Methods: A cross-sectional study was conducted on 5517 women having a birth history, from the Ethiopian Mini Demographic and Health Survey (EMDHS) 2019 dataset. Data management and analysis utilized STATA version 17, and R version 4.2.2. We employed chi-square test of association for the association between the predictor and the outcome variables, and also multilevel bivariate analysis to identify the significant factors of the outcome variables.<br>Results: Out of 5517 women with birth history, 2,842 (51.5%) were delivered at home, and also 269 (4.87%) experienced at least one U5M. As shown in the joint and marginal probabilities of home delivery and U5M, there is a significant association (χ^2 = 3.83, P-value < 0.0001). The odds ratio (OR = 2.19, 95% CI: 1.89–2.62) indicates that children born at home are 2.19 times more likely to die before age five, emphasizing the protective effect of institutional deliveries against child mortality.<br>Conclusion: Overall, home delivery and U5M are strongly influenced by a combination of individual, household, and community factors. Education, wealth, antenatal and postnatal care, and media exposure serve as protective elements, while rural residence, too younger or too older maternal age, and lower economic status increase the risks. Regional disparities further indicate the need for targeted interventions. Expanding healthcare access in rural areas, improving female education, promoting antenatal and postnatal care, and increasing media-based health awareness campaigns are essential steps to reducing home deliveries and under-five mortality in Ethiopia.
**Order of Authors:**	Wondaya Fenta Zewdia, MSC in Biostatistics
	Maru Zewdu Kassie, MSc in Biostatistics
	Melkamu Ayana Zeru
	Asteray Assmie Ayenew
**Opposed Reviewers:**	
**Additional Information:**	
**Question**	**Response**
**Financial Disclosure**<br><br>Enter a financial disclosure statement that	The author(s) received no specific funding for this work.

Powered by Editorial Manager® and ProduXion Manager® from Aries Systems Corporation

describes the sources of funding for the work included in this submission. Review the submission guidelines for detailed requirements. View published research articles from *PLOS ONE* for specific examples.<br><br>This statement is required for submission and **will appear in the published article** if the submission is accepted. Please make sure it is accurate.<br><br>**Funded studies**<br>Enter a statement with the following details:<br>• Initials of the authors who received each award<br>• Grant numbers awarded to each author<br>• The full name of each funder<br>• URL of each funder website<br>• Did the sponsors or funders play any role in the study design, data collection and analysis, decision to publish, or preparation of the manuscript?<br><br>Did you receive funding for this work?	
**Competing Interests**<br><br>Use the instructions below to enter a competing interest statement for this submission. On behalf of all authors, disclose any competing interests that could be perceived to bias this work—acknowledging all financial support and any other relevant financial or non-financial competing interests.<br><br>This statement is required for submission and **will appear in the published article** if the submission is accepted. Please make sure it is accurate and that any funding sources listed in your Funding Information later in the submission form are also declared in your Financial Disclosure statement.<br><br>View published research articles from *PLOS ONE* for specific examples.	The authors declare that they have no competing interests.

**NO authors have competing interests**<br><br>Enter: *The authors have declared that no competing interests exist.*<br><br>**Authors with competing interests**<br><br>Enter competing interest details beginning with this statement:<br><br>*I have read the journal's policy and the authors of this manuscript have the following competing interests: [insert competing interests here]*<br><br>* typeset	
**Ethics Statement**<br><br>Enter an ethics statement for this submission. This statement is required if the study involved:<br><br>• Human participants<br>• Human specimens or tissue<br>• Vertebrate animals or cephalopods<br>• Vertebrate embryos or tissues<br>• Field research<br><br>Write "N/A" if the submission does not require an ethics statement.<br><br>General guidance is provided below. Consult the submission guidelines for detailed instructions. **Make sure that all information entered here is included in the Methods section of the manuscript.**	All methods were conducted following the guidelines of the Demographic and Health Survey (DHS) program. Informed consent was waived by the International Review Board of DHS program data archivists. Upon submission of the consent paper to the DHS Program, a letter of permission to download the dataset for this study was obtained. The data were solely used for this study and could not be shared with other researchers without DHS consent. The methods adhered to the ethical standards of the Declaration of Helsinki.

Powered by Editorial Manager® and ProduXion Manager® from Aries Systems Corporation

**Format for specific study types**<br><br>**Human Subject Research (involving human participants and/or tissue)**<br>• Give the name of the institutional review board or ethics committee that approved the study<br>• Include the approval number and/or a statement indicating approval of this research<br>• Indicate the form of consent obtained (written/oral) or the reason that consent was not obtained (e.g. the data were analyzed anonymously)<br><br>**Animal Research (involving vertebrate animals, embryos or tissues)**<br>• Provide the name of the Institutional Animal Care and Use Committee (IACUC) or other relevant ethics board that reviewed the study protocol, and indicate whether they approved this research or granted a formal waiver of ethical approval<br>• Include an approval number if one was obtained<br>• If the study involved *non-human primates*, add *additional details* about animal welfare and steps taken to ameliorate suffering<br>• If anesthesia, euthanasia, or any kind of animal sacrifice is part of the study, include briefly which substances and/or methods were applied<br><br>**Field Research**<br><br>Include the following details if this study involves the collection of plant, animal, or other materials from a natural setting:<br>• Field permit number<br>• Name of the institution or relevant body that granted permission	
**Data Availability**<br><br>Authors are required to make all data underlying the findings described fully available, without restriction, and from the time of publication. PLOS allows rare exceptions to address legal and ethical concerns. See the PLOS Data Policy and FAQ for detailed information.	Yes - all data are fully available without restriction

A Data Availability Statement describing where the data can be found is required at submission. Your answers to this question constitute the Data Availability Statement and **will be published in the article**, if accepted.<br><br>**Important:** Stating 'data available on request from the author' is not sufficient. If your data are only available upon request, select 'No' for the first question and explain your exceptional situation in the text box.<br><br>Do the authors confirm that all data underlying the findings described in their manuscript are fully available without restriction?	
**Describe where the data may be found in full sentences. If you are copying our sample text, replace any instances of XXX with the appropriate details.**<br><br>• If the data are **held or will be held in a public repository**, include URLs, accession numbers or DOIs. If this information will only be available after acceptance, indicate this by ticking the box below. For example: *All XXX files are available from the XXX database (accession number(s) XXX, XXX.*).<br>• If the data are all contained **within the manuscript and/or Supporting Information files**, enter the following: *All relevant data are within the manuscript and its Supporting Information files.*<br>• If neither of these applies but you are able to provide **details of access elsewhere**, with or without limitations, please do so. For example:<br><br>*Data cannot be shared publicly because of [XXX]. Data are available from the XXX Institutional Data Access / Ethics Committee (contact via XXX) for researchers who meet the criteria for access to confidential data.*<br><br>*The data underlying the results presented in the study are available from (include the name of the third party*	The data underlying the findings of this study are publicly available from the DHS Program website. The data can be accessed at https://dhsprogram.com/data/ after free registration and approval of the data access request.

Powered by Editorial Manager® and ProduXion Manager® from Aries Systems Corporation

and contact information or URL).<br>• This text is appropriate if the data are owned by a third party and authors do not have permission to share the data.<br><br>* typeset	
Additional data availability information:	

Powered by Editorial Manager® and ProduXion Manager® from Aries Systems Corporation

Maru Zewdu Kassie
Department of Statistics, Assosa University
Assosa, Ethiopia
Email: maruzewdu68@gmail.com

Date: August 4, 2025

To: The Editor
PLOS ONE

Subject: Manuscript Submission: "Exploring the association between home delivery and under-five mortality in Ethiopia: A bivariate multilevel mixed-effects analysis"

Dear Editor,

We are pleased to submit our manuscript entitled "Exploring the association between home delivery and under-five mortality in Ethiopia: A bivariate multilevel mixed-effects analysis" for consideration for publication in *PLOS ONE*.

This study addresses a critical public health concern in Ethiopia (under-five mortality) by examining its association with home delivery practices. Utilizing data from the 2019 Ethiopian Mini Demographic and Health Survey (EMDHS), we applied a bivariate multilevel mixed-effects modeling approach to simultaneously analyze the determinants of home delivery and under-five mortality (U5M). Our findings show that children born at home are over twice as likely to die before age five, highlighting the crucial role of maternal healthcare access, education, and media exposure in improving child survival.

We believe this original and unpublished work offers meaningful insights into maternal and child health in low-resource settings, aligning with *PLOS ONE*'s mission to advance accessible, impactful science. All authors approve the manuscript and declare no conflicts of interest. We kindly request its consideration for publication and welcome any feedback during the review process. Thank you for your time and consideration.

Sincerely,

Maru Zewdu Kassie (Corresponding Author)
On behalf of all co-authors
Department of Statistics, Assosa University
Email: maruzewdu68@gmail.com
ORCID: https://orcid.org/0000-0001-5291-9623

Manuscript

Click here to access/download;Manuscript;Bivariate manuscript.docx

Exploring the association between home delivery and under-five mortality in Ethiopia: A bivariate multilevel mixed-effects analysis

Wondaya Fenta Zewdia[1*], Maru Zewdu Kassie[2*], Melkamu Ayana Zeru[1], Asteray Assmie Ayenew[3,4]

1 Department of Statistics, Bahir Dar University, Bahir Dar, Ethiopia

2 Department of Statistics, Assosa University, Assosa, Ethiopia

3 Department of Midwifery, Bahir University College of Medicine and Health Science, Bahir Dar, Ethiopia.

4 Department of Obstetrics and Gynaecology, Monash University, Melbourne, Victoria, Australia.

Corresponding Author: * maruzewdu68@gmail.com & handintouchto@gmail.com

Abstract

Background of the study: Child mortality remains a major public health issue in Ethiopia, with under-five mortality rates among the highest in sub-Saharan Africa. Despite efforts to improve maternal and child health, many women still give birth at home without skilled assistance, increasing the risk of neonatal and child mortality due to childbirth complications, inadequate postnatal care, and infections. So, this study aimed to investigate the association and determinants of home delivery and under-five mortality (U5M) using a multilevel bivariate modeling approach.

Methods: A cross-sectional study was conducted on 5517 women having a birth history, from the Ethiopian Mini Demographic and Health Survey (EMDHS) 2019 dataset. Data management and analysis utilized STATA version 17, and R version 4.2.2. We employed chi-square test of association for the association between the predictor and the outcome variables, and also multilevel bivariate analysis to identify the significant factors of the outcome variables.

Results: Out of 5517 women with birth history, 2,842 (51.5%) were delivered at home, and also 269 (4.87%) experienced at least one U5M. As shown in the joint and marginal probabilities of home delivery and U5M, there is a significant association ($\chi^2 = 3.83$, P-value < 0.0001). The odds ratio (OR = 2.19, 95% CI: 1.89–2.62) indicates that children born at home are 2.19 times more likely to die before age five, emphasizing the protective effect of institutional deliveries against child mortality.

Conclusion: Overall, home delivery and U5M are strongly influenced by a combination of individual, household, and community factors. Education, wealth, antenatal and postnatal care, and media exposure serve as protective elements, while rural residence, too younger or too older maternal age, and lower economic status increase the risks. Regional disparities further indicate the need for targeted interventions. Expanding healthcare access in rural areas, improving female education, promoting antenatal and postnatal care, and increasing media-based health awareness campaigns are essential steps to reducing home deliveries and under-five mortality in Ethiopia.

Key words: Home delivery, Under-five mortality, bivariate multilevel analysis, Women, Health Facility Births

Introduction

Child mortality remains a major global public health issue, with four million deaths occurring in the first month of life annually (1). U5M, the risk of death between birth and age five, is a key indicator of societal well-being (2). The U5M rate is a critical target of the Millennium Development Goals (MDGs) and Sustainable Development Goals (SDGs) (3). The SDGs aim to reduce U5M to at least 25 deaths per 1,000 live births by 2030, emphasizing the importance of maternal health facilities in achieving this goal (4). While the MDG 4 target of reducing child mortality by two-thirds was not fully met globally, certain regions, including East Asia and the Pacific, Latin America and the Caribbean, and 62 countries, made significant progress (5, 6).

Home delivery, prevalent in developing countries, is a significant contributor to U5M (7). Despite improvements in health interventions, Sub-Saharan Africa continues to experience high under-five mortality rates (8). Global health policies stress institutional deliveries to reduce U5M, but neonatal mortality decreases at a slower pace compared to child mortality, particularly in Sub-Saharan Africa (9, 10). According to the 2022 World Health Organization (WHO) report, sub-Saharan Africa accounted for 57% of global U5M, with an estimated 2.8 million deaths (95% CI: 2.5–3.3 million), despite representing only 30% of total live births worldwide. The region also exhibited the highest neonatal mortality rate globally, with 27 deaths per 1,000 live births, followed by Central and Southern Asia, which reported a neonatal mortality rate of 21 deaths per 1,000 live births (11). The region accounts for 55% of global U5M, with East Africa having the highest infant mortality rate (92.2 deaths per 1,000 live births) (12). In Ethiopia, the prevalence of home deliveries declined from 73% in 2016 to 51% in 2019. However, a significant proportion of births continue to occur at home, which may be a key contributing factor to the persistently high under-five mortality (U5M) rate in the country (13). This trend suggests a substantial burden on child health, as home deliveries are often associated with inadequate medical care, increased risk of complications, and higher neonatal and infant mortality rates.

Previous studies have identified multiple factors influencing childhood mortality, including socioeconomic conditions, maternal and child-related determinants, and healthcare utilization (14, 15, 16, 17, 18). Based on the research (19), the most common causes of under-five mortality include acute respiratory infections, diarrhea, malaria, and birth complications, with neonatal

disorders and infections remaining significant contributors in low- and middle-income countries (LMICs). Another study revealed that low parental education, younger maternal age, short birth intervals, non-immunized children, and inadequate toilet facilities increase the risk of child mortality, while access to safe healthcare reduces it (14). The association between home births and U5M remains a significant public health concern, as delivery location influences neonatal and child survival outcomes (20, 21, 22). Home births are often linked to higher mortality due to limited access to skilled care and emergency interventions (23, 24). Despite the decline in home deliveries in Ethiopia, they still account for a substantial proportion of births, potentially driving the persistently high U5M rate (25, 26). However, to date, rare comprehensive ~~study has~~ examined this relationship using ~~a robust multilevel analytical approach~~ in Ethiopia.

Our study addresses this gap by employing a **bivariate multilevel analysis** using data from the EMDHS 2019. Unlike previous studies that used enumeration areas as the second-level unit in multilevel modeling, we adopt **zones** as a more policy-relevant and interpretable level of analysis. This approach accounts for the correlated nature of binary outcomes, enabling a more precise investigation of the association between home delivery and U5M. Given Ethiopia's unique demographic profile, characterized by high birth and mortality rates, our findings will provide valuable insights for targeted interventions aimed at reducing child mortality. Ultimately, our study seeks to inform strategies that enhance maternal and child health, with the overarching goal of lowering U5M prevalence in Ethiopia.

Materials and methods

Study area, data sources, and study population

This study was conducted in Ethiopia, the second most populous country in Africa, with over 110 million inhabitants as of 2022. Ethiopia is administratively divided into two chartered cities and nine regional states, which are further subdivided into zones, woredas, and kebeles. The study utilized cross-sectional, community-based data from the EMDHS 2019, collected by the Ethiopian Public Health Institute in collaboration with the Federal Ministry of Health and the Central Statistical Agency (CSA). A two-stage stratified cluster sampling method was employed. In the first stage, 305 enumeration areas were selected based on the 2019 Ethiopian Population

and Housing Census (EPHC) frame, with 93 urban and 212 rural clusters sampled proportionally. In the second stage, 30 households were randomly selected from each cluster.

Data were extracted from the Kids Record (KR) files, which contain maternal and child health information, focusing on variables related to insufficient dietary diversity (27). Ethiopia has conducted four full-scale Demographic and Health Surveys (DHS) in 2000, 2005, 2011, and 2016, along with two Mini DHS surveys in 2014 and 2019. The 2019 EMDHS, conducted between March 21 and June 28, 2019, serves as the data source for this study. We accessed the anonymized dataset from the DHS Program website **https://dhsprogram.com/data/** on April 4, 2024. The data were de-identified before access, and the authors had no access to any information that could identify individual participants during or after data collection.

Inclusion and Exclusion Criteria

This study included women aged 15–49 years who were married and had at least one live birth within five years prior to the survey. Additionally, only children under five with complete data on under-five mortality (U5M) and place of delivery were considered. Participants with incomplete or missing information on key variables were excluded from the analysis.

Study Variables

The response variables

The primary outcome variables in this study were U5M and home delivery, both defined according to the EMDHS 2019. U5M was categorized as a binary variable, indicating whether a child died before reaching their fifth birthday (target) or survived. Similarly, place of delivery was classified as either home delivery (target) or health facility delivery.

Explanatory variables

The study included a range of individual- and community-level explanatory variables for both outcome measures. These included:

- **Child-related factors**: Age (in months), sex, and twin status.

- **Maternal factors**: Current age, marital status, education level, contraceptive use, antenatal care follow-up, postnatal care, health worker counseling during pregnancy, and last birth by cesarean section.
- **Household factors**: Wealth index, household head's sex, and access to mass media.
- **Community-level factors**: Region, place of residence, community education level, community wealth index, media exposure, and antenatal visitation

Statistical Analysis

Joint Multilevel Analysis

In order to model the relationships between two correlated binary responses with one or more covariates, multilevel joint models one of the families of multivariate models take into account the covariates that are measured at different levels of a hierarchical structure and how the interactions between covariates that are measured at different levels affect the response variables (28, 29, 30, 31, 32). The Multilevel binary having two-level model is given by;

$$logit(\pi_{ij}) = log\left(\frac{\pi_{ij}}{1-\pi_{ij}}\right) = \gamma_{00} + \gamma_{k0}X_{kij} + \gamma_{0q}Z_{qj} + U_{0j} + U_{kj}U_{kj}\ .\ .\ . \qquad (1)$$

Where γ_{00} is the average regression intercept, X_{kij} represent the k level-one covariates, and Z_{qj} represent the q level-two covariates, whereas, γ_{k0} and γ_{0q} are the corresponding regression coefficients for level-one and level-two explanatory variables, U_{0j} and U_{kj} are the random effect of the model parameters at level two. It is expected that the vector of random effects $U_{0j}, U_{1j}, U_{2j}, \dots, \dots, \dots, U_{kj}$ follow a normal distribution with mean zero and variance σ^2_{u0}. Akaike's information criterion (AIC) and the Bayesian information criterion (BIC) determine the best multilevel model out of multilevel models. More basically to determine a measure of the similarity between two individuals from the same neighborhood we apply intra-class correlation (ICC). The value of ICC was ~~take~~ between 0 and 1 inclusive. Basically ICC was used to determine the proportion of cluster-level variance compared to the total variance that expresses the proportion of the total variance at the cluster level. Each and every U_{0j} and σ^2_{u0} would be zero if there was no variation among the area effects. Multilevel analysis is inappropriate for further data analysis when the ICC ~~was~~ become zero. The intercept- only model decomposes the variance into two independent components. We know that $Y_{ij} = \gamma_{00} + U_{0j} + \epsilon_{ij}$ from

this $var(Y_{ij}) = var(U_{0j}) + var(\epsilon_{ij}) = \sigma_{u0}^2 + \sigma_e^2$. Covariance between two individuals ($i \neq j$) in the same group: $cov(Y_{ij}, Y_{i'j}) = var(U_{0j}) = \sigma_{u0}^2$.

Using this model, we can define the ICC by the equation:

$$\boldsymbol{ICC = \rho(Y_{ij}, Y_{i'j}) = \frac{cov(Y_{ij},Y_{i'j})}{var(U_{0j})+var(\epsilon_{ij})} = \frac{\sigma_{u0}^2}{\sigma_{u0}^2+\sigma_e^2} = \frac{\sigma_{u0}^2}{\sigma_{u0}^2+\frac{\pi^2}{3}}} \qquad (\mathbf{2})$$

Where, σ_e^2 is the variance of individual level units and σ_{u0}^2 is the variance of the higher-level residual errors. In the multilevel logit model, the estimated level-1 variance is the standard logistic distribution, $\pi^2/3$ which is considered as level-1 residual variance(σ_e^2), $\boldsymbol{i.e}$ $\sigma_e^2 = \pi^2/3 \approx 3.29$. In the absence of a multilevel structure, a single-level individual analysis is appropriate. Then there is a proportional change in variance (PCV) or coefficient determination (R_1^2), that shows how much the proportion of variance is explained by level-1 and level-2 variables. For the proportion of variance explained at the level-1, is determined as:

$$PCV = R_1^2 = \frac{\sigma_{e|b}^2 - \sigma_{e|m}^2}{\sigma_{e|b}^2} \qquad (3)$$

Where $\sigma_{e|b}^2$ is the lowest level residual variance for the baseline model, which is the intercept only model, and $\sigma_{e|m}^2$ is the lowest residual variance for the comparison model while the proportion of variance explained at the community level, is determined as:

$$R_2^2 = \frac{\sigma_{u0|b}^2 - \sigma_{u0|m}^2}{\sigma_{u0|b}^2} \qquad (4)$$

Where, $\sigma_{u0|b}^2$ is the highest-level residual variance for the baseline is model and $\sigma_{u0|m}^2$ is the highest-level residual variance for the comparison model.

Let Y_1 and Y_2 be home delivery and U5M of this study, then the two bivariate binary responses denoted as a vector of Y = $(\gamma_{11}\ \gamma_{01}\ \gamma_{10}\ \gamma_{00}]^T$ and structured as (Table 1).

Table 1: The joint probability of the response variables

	U5M(Y_2)		
Home Delivery(Y_1)	1	0	Total

1	Υ_{11}	Υ_{10}	Υ_{1}
0	Υ_{01}	Υ_{00}	$1-\Upsilon_{1}$
Total	Υ_{2}	$1-\Upsilon_{2}$	1

Moreover, the joint multilevel model can be written as follows the first two equation as marginal of single out come variable and last equation indicate joint equation simultaneously;

$$g_1(x) = logit\left(\pi_1(x)\right) = \beta_1 x^T,$$

$$g_2(x) = logit\left(\pi_2(x)\right) = \beta_2 x^T$$

$$g_3(x) = lnln\psi(x) = \beta_3 x^T \qquad (5)$$

The odds ratio (OR), which is used to ~~describes~~ the dependence of home delivery and U5M using basic link function of logit stated as;

$$\Psi = \frac{Pr(Y2 = 1 \mid Y1 = 1)}{Pr(Y2 = 1 \mid Y1 = 0)} = \frac{\pi_{00}\,\pi_{11}}{\pi_{01}\,\pi_{10}}, \quad \text{where } \Psi \geq 0. \qquad (6)$$

Where $x = [1\ x_1 x_2 \cdots x_k]^T$, is a vector of the covariate, $\beta_1{}^T = [\beta_{01}\ \ \beta_{11}\ \beta_{12} \cdots \beta_{1K}]$, $\beta_2{}^T = [\beta_{02}\ \beta_{12}\ \beta_{22} \cdots \beta_{2K}]$, and $\beta_3{}^T = [\beta_{03}\ \ \beta_{13}\ \beta_{23} \cdots \beta_{K3}]$ are the parameters, $\pi_1(x)$ is the marginal probability function of Y_1, $\pi_2(x)$ is the marginal probability function of Y_2, and $\Psi(x)$ is the odds ratio that shows the association between Y_1 and Y_2 Erro[illegible]Bookmark not defined. The null hypothesis H_0: $\beta_{11} = \beta_{10}$ can be tested for independence in the presence of covariates between the two binary outcome variables Y_1 and Y_2 (29, 30). The Newton-Raphson algorithm iterative procedure was used to apply the maximum likelihood method to obtain the model's parameters estimator (30, 33).

Results

Table 2 presents the distribution of various independent variables (covariates) and their association with home delivery and U5M in Ethiopia. The chi-square (χ^2) p-values indicate the statistical significance of the association between each independent variable and the two outcome variables (home delivery and U5M). A p-value less than 0.05 suggest a statistically significant association. Out of 5517 women with birth history, 2,842 (51.5%) were delivered at home, and also 269 (4.87%) experienced at least one U5M.

Younger mothers (15-18 years) had a lower proportion of home deliveries compared to older mothers, and also the highest proportion of U5M was found among children born to older mothers (35-49 years). Additionally, the association of ~~mothers~~ age with both home delivery and U5M is statistically significant (p-value<0.0001). Religion also influences home delivery and U5M, with Muslim mothers showing the highest proportion of both outcomes. Place of residence was also another important variable. Rural mothers had a much higher proportion of home deliveries (86%) compared to urban mothers (14%). Also U5M was also higher in rural residents (83.6%). As it is viewed in the chi-square p-value (p-value<0.0001), mother's residence is highly associated with the two outcomes.

Regarding education, Women with no education had the highest proportion of home deliveries (68.8%), also U5M was also highest among children born to uneducated mothers. Higher education levels significantly reduce both home delivery and U5M (p < 0.0001). Also, wealth index influences home delivery and U5M, with poorer households ~~had~~ a higher prevalence of home delivery (62.2%) and U5M (55.8%). ~~That is the wealthiest households had the lowest home delivery and U5M rates.~~ This association is significant (P-value<0.0001).

Sex of the household head also plays a crucial role. Households headed by males had a slightly higher home delivery rate (77.1%) and U5M (81.4%). This association is statistically significant (p < 0.0001). Regarding media exposure, mothers with media exposure had significantly lower home delivery (21.6%) and U5M (29%). Those with no media exposure had higher home delivery (78.4%) and U5M (71%). Also, Media exposure is significantly associated with both outcomes (p < 0.0001).

Healthcare access and utilization significantly impact both outcomes. Women who receive antenatal care (ANC) visits, counseling from health workers, and postnatal check-ups within two months have lower home delivery and U5M rates. Conversely, those lacking these services exhibit higher risks. For instance, women who received pregnancy counseling had significantly lower home delivery (21.8%) and U5M (22.4%). Lack of counseling led to a higher risk of both. Contraceptive use is another influential factor, with non-users having higher home delivery and U5M rates.

Regarding community-level factors, low community ANC visit, low wealth index, low education level, and low media exposure were significantly associated with higher home delivery and U5M. These associations were all statistically significant ($p < 0.0001$). In conclusion Table 2 highlights that socioeconomic, demographic, and healthcare-related factors significantly influence home delivery and under-five mortality in Ethiopia. Key risk factors include low maternal education, rural residence, poverty, lack of ANC visits, lack of health counseling, short birth intervals, and lack of media exposure. Addressing these factors through improved maternal healthcare access and education can help reduce home deliveries and under-five mortality.

Figure 1 depicted the relationship between home delivery and U5M. U5M accounted for 43% of all births at home, whereas they made up 57.2% of births in health facilities. Compared to home births (49%), hospital births had a significantly better survival rate (51.2%). Despite showing proportions, this figure emphasizes how home births are associated with increased risks of U5M because of insufficient newborn care and a lack of professional birth attendance.

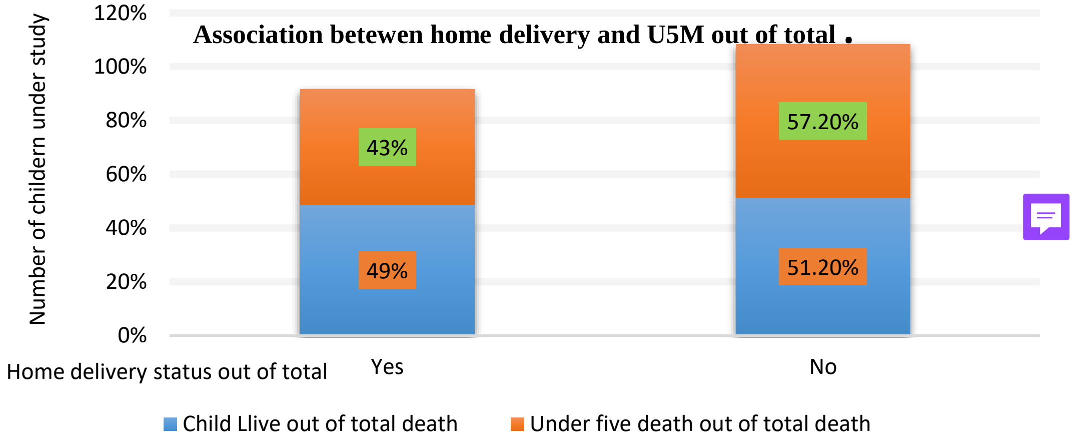

Figure 1: Association between home delivery and U5M out of total da[illegible]nder study

Joint and marginal probability of home delivery and U5M

Table 3 presents the joint and marginal probabilities of home delivery and U5M, along with their association and odds ratio. The chi-square test ($\chi^2 = 3.83$, P-value < 0.0001) indicates a statistically significant relationship between home delivery and U5M, suggesting that the place

of delivery impacts child survival. Regarding the odds ratio, the odds ratio (OR = 2.19, 95% CI: 1.89–2.62) indicates that children born at home have 2.19 times higher odds of dying before the age of five compared to those born in a health facility. Since the confidence interval (CI) does not include 1, the association is statistically significant. This reinforces the idea that institutional deliveries are protective against child mortality, likely due to better medical care, skilled birth attendance, and postnatal support.

Model comparison

Table 4 presents the model comparison results for four different models (Model I to Model IV) based on variance, ICC, PCV, and model selection criteria (AIC and BIC) for both outcome variable at a time. The Pearson chi-square/DF values are also provided to assess the goodness of fit. In conclusion, among the four models, Model IV appears to be the best fit, as it has the lowest variance, the highest PCV (explaining 54% of the variance), and the smallest AIC and BIC values. The ICC value of 0.21 suggests that household-level factors significantly contribute to the variability in the outcome. The Pearson chi-square/DF values indicate that all models fit well, with Model II showing an exact value of 1. However, Model IV is preferred overall due to its superior explanatory power and improved model fit together for both ~~out come~~ at same time. As of bivariate analysis we ~~were build~~ single model analysis for both outcome variables simultaneously.

Parameter estimates of the multilevel bivariate modeling of home delivery and U5M

Table 5 presents the results of a multilevel bivariate model analyzing the association between various socio-demographic and household-level factors with home delivery and U5M. The table includes estimated coefficients (Estimate), adjusted odds ratios (AOR), and 95% confidence intervals (CI) for the estimate of each factor.

Most importantly as of bivariate multilevel the overall model odd ratio is greater than one [AOR (95% CI) =3.501 (1.267, 6.713)]. This result shows that there were strong positive statistical ~~association~~ between home delivery and U5M. This shows that home delivery was ~~positive strong~~ for under five mortality in Ethiopia. Results were developed by loop rule by using both of the outcome variables at a time by considering all independent ~~variable~~. The negative intercept (-

0.4113) with an AOR of 0.662 for both home delivery and U5M suggests that, when all other variables are at their reference category, the odds of home delivery and U5M are lower.

Maternal age also influences both home delivery and U5M, as mothers aged 15-18 had 1.699 times higher odds of delivering at home and 2.075 times higher odds of U5M compared to the reference group (19–34 years). Additionally, mothers aged 35–49 had slightly elevated odds of home delivery (1.185) and U5M (1.350), though weaker compared to the youngest group. Mothers place of residence also have a crucial role for home delivery and U5M. As of too young or to old be of mother were aggravated the problem of home delivery and U5M. Mothers who follow orthodox religion were increase the risk of home delivery and U5M compared to other religion in Ethiopia. Similarly mother who follow Muslim religion were increase the problem of home delivery. Rural dweller mothers had 1.584 times higher odds of home delivery and 2.054 times higher odds of U5M compared to urban mothers, showing the impact of geographic access to healthcare services. Moreover residents come from rural dwellers were increase home delivery for mother and U5M for a child simultaneously.

Maternal education demonstrated a strong protective effect. Women with no formal education had 2.386 times higher odds of home delivery and 1.284 times higher odds of U5M compared to those with primary education. Higher education significantly reduced the odds of both home delivery (AOR = 0.445) and U5M (AOR = 0.506), emphasizing the importance of female education in improving maternal and child health outcomes. Importantly mothers have secondary education status reduce the risk of home delivery compared to primary. Most importantly mother who have an experience of contraceptive usage were reduce the problem of Home delivery and U5M compared to nonuser.

Regional disparities were also notable, with the highest odds of home delivery observed in Gambela (AOR = 27.66), Benishangul (AOR = 23.10), and Tigray (AOR = 22.42) similar for Amhara, Oromia, SNNPR regions. In terms of child mortality, Somalia (AOR = 8.248) and Dire Dawa (AOR = 2.247) exhibited the highest odds, highlighting differences in healthcare availability and utilization across Ethiopia compared to Addis Abeba. These regions exhibit the risk of home delivery and U5M simultaneously. Wealth index was another critical factor. Poor households had 1.377 times higher odds of home delivery and 1.185 times higher odds of U5M

compared to middle-income households. Additionally, Wealthier households had a lower risk of home delivery (AOR = 0.281) and U5M (AOR = 0.779), reinforcing the link between economic status and child health outcomes.

Media exposure also played a protective role, as women with media exposure had lower odds of home delivery (AOR = 0.913) and U5M (AOR = 0.932), indicating the influence of health awareness campaigns. Postnatal care attendance within two months significantly reduced both home delivery (AOR = 0.619) and U5M (AOR = 0.625), reinforcing the importance of follow-up care in reducing child mortality. Antenatal care (ANC) visits were another strong protective factor. Mothers with no ANC visits had significantly higher odds of home delivery (AOR = 0.[illegible]), highlighting the importance of prenatal checkups. Regarding the community-level factors, high levels of community ANC visits, wealth index, education, and media exposure were all associated with lower odds of home delivery and U5M, emphasizing the collective impact of community-level interventions. Having community (zones) level ~~variable~~ (wealth index, education level, media exposure and antenatal care) ~~were lower expedite~~ the risk of home delivery and U5M in Ethiopia concurrently.

Overall, this analysis highlights the need for targeted interventions such as expanding healthcare access in rural areas, increasing female education, promoting antenatal and postnatal care, and enhancing media-based health awareness campaigns to reduce home deliveries and under-five mortality in Ethiopia.

Discussion

This study examined the determinants of home delivery and under-five mortality (U5M) using a multilevel bivariate modeling approach. The findings highlight the significant influence of maternal, household, and community-level factors on both outcomes, emphasizing the need for targeted interventions to improve maternal and child health. The ~~result~~ revealed that, out of 5517 reproductive aged women who had been married and had at least one child born within the five years prior to the survey, 2,842 (51.5%) were delivered at home, and also 269 (4.87%) experienced at least one U5M.

The study revealed that delivery outside the health facility (home) increase the U5M. This finding aligns with previous researches (20, 34, 35, 36), as the hospital delivery saves new-born lives. This finding also align with (22), with their finding revealed that health facility delivery is found to reduce the risk of neonatal mortality by 29% in low and middle income countries. As we have seen the home delivery outcome and its determinants, 51.5% of births occurred at home, with rural residence, lower maternal education, and economic disadvantage being major contributing factors. Women in rural areas had significantly higher odds of delivering at home compared to their urban counterparts, likely due to limited healthcare access, poor infrastructure, and cultural preferences. This finding aligns with previous studies in Ethiopia (23, 37, 38, 39) and other low-income countries (40, 41), where distance to healthcare facilities and transportation challenges remain barriers to institutional delivery.

Maternal education emerged as a strong determinant, with higher education significantly reducing the odds of home delivery and U5M. Educated women are more likely to seek healthcare services, be aware of birth risks, and have better decision-making autonomy. This finding is aligns with (37, 39, 42, 43). In their findings, the home delivery rate and under-five mortality (U5M) rate among educated mothers were lower compared to uneducated mothers. Similarly, wealth status played a crucial role, as poorer households had higher odds of home delivery and U5M compared to wealthier ones, reinforcing the financial burden of facility-based deliveries. These findings underscore the importance of expanding maternal education programs and addressing economic barriers to ensure safe deliveries. This association is well-documented in the literature, where lower wealth indices correlate with increased under-five mortality rates (23, 39, 43, 44). In their findings, economic constraints often limit access to quality healthcare and nutrition, contributing to higher mortality.

The utilization of ANC services and postnatal check-ups significantly reduces the odds of home delivery and U5M. Mothers attending four or more ANC visits are more likely to deliver in health facilities and have better child survival rates. This finding is supported by research indicating that adequate ANC visits are associated with reduced under-five mortality (45, 46). In their findings, antenatal care visit decreases the likelihood of under-five mortality. Additionally, our study identifies significant regional disparities in home delivery practices and U5M rates across Ethiopia. Regions such as Afar, Gambela, Somali, and Benishangul-Gumuz exhibit higher

odds of home delivery and U5M compared to Addis Ababa. These variations may stem from differences in healthcare infrastructure, cultural practices, and socioeconomic factors. Similar regional disparities have been reported in other studies, underscoring the need for region-specific interventions (23, 39).

The insights from this study underscore the necessity for targeted interventions to reduce home deliveries and under-five mortality in Ethiopia. Strategies should focus on enhancing maternal education, improving economic conditions, and increasing access to quality antenatal and postnatal care, particularly in rural and underserved regions. Addressing these determinants holistically can contribute to achieving national and global child survival goals. In conclusion, our research highlights the multifaceted factors influencing home delivery practices and under-five mortality in Ethiopia. By aligning our findings with existing literature, we emphasize the critical areas for intervention to improve child health outcomes in the country.

Conclusion

Most importantly out of total under five ~~death~~ (269) in Ethiopia 115(43%) ~~death~~ were caused by home delivery related difficulties. This study highlights key association determinants of home delivery and U5M in Ethiopia simultaneously. Maternal age, place of residence, education, household wealth, ANC visits, and regional disparities significantly influence both outcomes ~~at a time~~ in Ethiopia. Children born to older mothers, those in rural areas, and those from poorer households face higher U5M risks, while higher maternal education, ANC visits, and urban residence serve as protective factors. Areas with more health facility deliveries than ~~health facility~~ deliveries have reduced under five mortality. Maximizing delivery in health facility while improving quality across the health system may help to reduce high under five mortality.

This study ~~conclude~~ that there were strong positive statistical ~~association~~ between home delivery and U5m. ~~This~~ shows that home delivery ~~was positive strong for~~ under five mortality in Ethiopia. Regional disparities further emphasize the need for targeted, location-specific interventions. Strengthening maternal health services, expanding education, improving economic conditions, and promoting ANC utilization are crucial in reducing home deliveries and U5M. A multifaceted, evidence-based approach is essential to improving maternal and child health

outcomes, supporting Ethiopia's progress toward national and global child survival goals. This has important ramifications for public policy, especially when it comes to combining maternal and child health services with family education. The study's conclusions indicate that in order to lower child mortality, the Ethiopian government and interested parties must work together to prevent home births and deaths of children under five, increase public awareness, and offer community education. As of prevalence of under-five mortality was still high in Ethiopia, ~~integrated task force must be look the root cause and confounding factor~~.

Strength and Limitations

This study has strengths and limitations. One of its key strengths is the use of a large, nationally representative dataset, enhancing the generalizability of the findings to the Ethiopian population. Additionally, the application of multilevel modeling allows for a more comprehensive analysis by accounting for both individual- and household-level variations. The study also provides critical insights into the regional disparities in home delivery and U5M, highlighting the need for targeted interventions.

However, certain limitations should be acknowledged. As a cross-sectional study, it establishes associations but cannot determine causality. The reliance on self-reported data may introduce recall bias. Additionally, some potential predictor variables such as the child's birth weight, clinical variables, and maternal health conditions like diabetes mellitus and hypertension were not collected. Moreover, we only considered data from 64 Ethiopian administrative zones, as data from 8 zones were not included in the 2019 EMDHS data collection. Despite these limitations, the study provides valuable evidence to inform maternal and child health policies, emphasizing the need for region-specific strategies to improve child survival outcomes. Therefore, we recommend that future studies on maternal and child health be conducted by incorporating data from all Ethiopian zones and including potential variables that could not be included in this study, once the EDHS releases data encompassing these factors.

Acknowledgements
Our special thanks go to the DHS Program for assisting us in accessing the EMDHS dataset along with its GPS.

Declarations

Ethics approval and consent to participate

All methods were conducted following the guidelines of the Demographic and Health Survey (DHS) program. Informed consent was waived by the International Review Board of DHS program data archivists. Upon submission of the consent paper to the DHS Program, a letter of permission to download the dataset for this study was obtained. The data were solely used for this study and could not be shared with other researchers without DHS consent. The methods adhered to the ethical standards of the Declaration of Helsinki.

Consent for publication

Not applicable

Availability of data and materials

The data underlying the findings of this study are publicly available from the DHS Program website. The data can be accessed at **https://dhsprogram.com/data/** after free registration and approval of the data access request.

Competing interests

The authors declare that they have no competing interests.

Funding

The authors didn't get specific fund for this study.

Authors' contributions

WFZ and MZK conceptualized the study, prepared the manuscript, analyzed the data, interpreted the final results, and thoroughly revised the manuscript. MAZ and AAA contributed to data analysis, supervision, and revision. Finally, all authors read and approved the final manuscript.

Reference:

1. Lawn JE, Cousens S, Zupan J. 4 million neonatal deaths: when? Where? Why? The lancet. 2005;365(9462):891-900.
2. UNICEF. The State of the World's Children 2006: Excluded and Invisible', 2005. UNICEF: New York. 2016.
3. Unicef. Under-five mortality. 2020. 2021.

4. Dheresa M, Roba HS, Daraje G, Abebe M, Tura AK, Yadeta TA, et al. Uncertainties in the path to 2030: Increasing trends of under-five mortality in the aftermath of Millennium Development Goal in Eastern Ethiopia. Journal of global health. 2022;12.
5. Bexell M, Jönsson K, editors. Responsibility and the United Nations' sustainable development goals. Forum for development studies; 2017: Taylor & Francis.
6. You D, Hug L, Ejdemyr S, Idele P, Hogan D, Mathers C, et al. Global, regional, and national levels and trends in under-5 mortality between 1990 and 2015, with scenario-based projections to 2030: a systematic analysis by the UN Inter-agency Group for Child Mortality Estimation. The Lancet. 2015;386(10010):2275-86.
7. Chowdhury AH, Islam S, Abdul Karim D. Covariates of neonatal and post-neonatal mortality in Bangladesh. Global J Hum Soc Sci Res. 2013;13(4).
8. Liu L, Oza S, Hogan D, Chu Y, Perin J, Zhu J, et al. Global, regional, and national causes of under-5 mortality in 2000–15: an updated systematic analysis with implications for the Sustainable Development Goals. The Lancet. 2016;388(10063):3027-35.
9. Okeke EN, Chari AV. Can institutional deliveries reduce newborn mortality? Evidence from Rwanda. RAND Working Paper 1072: Citeseer; 2014.
10. Gaffan N, Kpozehouen A, Degbey C, Ahanhanzo YG, Paraïso MN. Effects of household access to water, sanitation, and hygiene services on under-five mortality in Sub-Saharan Africa. Frontiers in public health. 2023;11:1136299.
11. Organization WH. Newborn Mortality: World Health Organization; 2022 [Available from: https://www.who.int/news-room/fact-sheets/detail/newborn-mortality.
12. Sharrow D, Hug L, You D, Alkema L, Black R, Cousens S, et al. Global, regional, and national trends in under-5 mortality between 1990 and 2019 with scenario-based projections until 2030: a systematic analysis by the UN Inter-agency Group for Child Mortality Estimation. The Lancet Global Health. 2022;10(2):e195-e206.
13. Indicators K. Mini demographic and health survey. EPHI and ICF. 2019.
14. Mondal MNI, Hossain MK, Ali MK. Factors influencing infant and child mortality: A case study of Rajshahi District, Bangladesh. Journal of Human Ecology. 2009;26(1):31-9.
15. Ekholuenetale M, Wegbom AI, Tudeme G, Onikan A. Household factors associated with infant and under-five mortality in sub-Saharan Africa countries. International Journal of Child Care and Education Policy. 2020;14:1-15.
16. Adedokun ST, Yaya S. Childhood morbidity and its determinants: evidence from 31 countries in sub-Saharan Africa. BMJ Global Health. 2020;5(10):e003109.
17. Baraki AG, Akalu TY, Wolde HF, Lakew AM, Gonete KA. Factors affecting infant mortality in the general population: evidence from the 2016 Ethiopian demographic and health survey (EDHS); a multilevel analysis. BMC pregnancy and childbirth. 2020;20:1-8.
18. Ayele BA, Abebaw Tiruneh S, Azanaw MM, Shimels Hailemeskel H, Akalu Y, Ayele AA. Determinants of under-five mortality in Ethiopia using the recent 2019 Ethiopian demographic and health survey data: nested shared frailty survival analysis. Archives of Public Health. 2022;80(1):137.
19. Tekin M. Under-Five Mortality Causes. Mortality rates in middle and low-income countries. 2022:37.
20. Das U, Chaplot B, Azamathulla HM. The role of place of delivery in preventing neonatal and infant mortality rate in India. Geographies. 2021;1(1):47-62.
21. Chaka EE, Mekurie M, Abdurahman AA, Parsaeian M, Majdzadeh R. Association between place of delivery for pregnant mothers and neonatal mortality: a systematic review and meta-analysis. European Journal of Public Health. 2020;30(4):743-8.
22. Tura G, Fantahun M, Worku A. The effect of health facility delivery on neonatal mortality: systematic review and meta-analysis. BMC pregnancy and childbirth. 2013;13:1-9.

23. Chernet AG, Dumga KT, Cherie KT. Home delivery practices and associated factors in Ethiopia. Journal of reproduction & infertility. 2019;20(2):102.
24. Zewudie AT, Gelagay AA, Enyew EF. Determinants of Under-Five Child Mortality in Ethiopia: Analysis Using Ethiopian Demographic Health Survey, 2016. International journal of pediatrics. 2020;2020(1):7471545.
25. Tariku M, Enyew DB, Tusa BS, Weldesenbet AB, Bahiru N. Home delivery among pregnant women with ANC follow-up in Ethiopia; Evidence from the 2019 Ethiopia mini demographic and health survey. Frontiers in Public Health. 2022;10:862616.
26. Teferi HM, San Sebastian M, Baroudi M. Factors associated with home delivery preference among pregnant women in Ethiopia: a cross-sectional study. Global Health Action. 2022;15(1):2080934.
27. Ephi I. Ethiopia mini demographic and health survey 2019: key indicators. Rockville, Maryland, USA: EPHI and ICF. 2019.
28. Sturrock HJ, Pullan RL, Kihara JH, Mwandawiro C, Brooker SJ. The use of bivariate spatial modeling of questionnaire and parasitology data to predict the distribution of Schistosoma haematobium in Coastal Kenya. PLoS neglected tropical diseases. 2013;7(1):e2016.
29. Gulshan J, Khan A, Islam MA. Modelling Correlated Bivariate Binary Data: A Comparative View. Bulletin of the Malaysian Mathematical Sciences Society. 2022;45(S1):251-70.
30. Purhadi P, Fathurahman M. A Logit Model for Bivariate Binary Responses. Symmetry. 2021;13(2):326.
31. Islam MA, Chowdhury RI, Briollais L. A bivariate binary model for testing dependence in outcomes. Bulletin of the Malaysian Mathematical Sciences Society. 2012;35(4):845-58.
32. Bhuyan MJ, Islam MA, Rahman MS. A bivariate Bernoulli model for analyzing malnutrition data. Health Services and Outcomes Research Methodology. 2018;18(2):109-27.
33. Liu B, Sze J, Li L, Ornstein KA, Taioli E. Bivariate Spatial Pattern between Smoking Prevalence and Lung Cancer Screening in US Counties. International journal of environmental research and public health. 2020;17(10).
34. Pal S. Impact of hospital delivery on child mortality: An analysis of adolescent mothers in Bangladesh. Social Science & Medicine. 2015;143:194-203.
35. Mekonnen Y, Tensou B, Telake DS, Degefie T, Bekele A. Neonatal mortality in Ethiopia: trends and determinants. BMC public health. 2013;13:1-14.
36. Kibret GD, Demant D, Hayen A. The effect of distance to health facility on neonatal mortality in Ethiopia. BMC Health Services Research. 2023;23(1):114.
37. Hailu D, Tadele H, Tadesse BT, Alemayehu A, Abuka T, Woldegebriel F, et al. Home delivery practice and its predictors in South Ethiopia. PloS one. 2021;16(8):e0254696.
38. Abate GT, Dereje M, Hirvonen K, Minten B. Geography of public service delivery in rural Ethiopia. World Development. 2020;136:105133.
39. Yemane GD. The factors associated with under-five mortality in Ethiopia. Annals of Medicine and Surgery. 2022;79:104063.
40. Kifle MM, Kesete HF, Gaim HT, Angosom GS, Araya MB. Health facility or home delivery? Factors influencing the choice of delivery place among mothers living in rural communities of Eritrea. Journal of Health, Population and Nutrition. 2018;37:1-15.
41. le R Booysen F. Urban–rural inequalities in health care delivery in South Africa. Development Southern Africa. 2003;20(5):659-73.
42. Gebremichael SG, Fenta SM. Under-Five Mortality and Associated Risk Factors in Rural Settings of Ethiopia: Evidences from 2016 Ethiopian Demographic and Health Survey. Advances in Public Health. 2020;2020(1):8430246.

43. Geremew BM, Gelaye KA, Melesse AW, Akalu TY, Baraki AG. Factors affecting under-five mortality in Ethiopia: a multilevel negative binomial model. Pediatric health, medicine and therapeutics. 2020:525-34.
44. Kiross GT, Chojenta C, Barker D, Tiruye TY, Loxton D. The effect of maternal education on infant mortality in Ethiopia: A systematic review and meta-analysis. PloS one. 2019;14(7):e0220076.
45. Fenta SM, Fenta HM. Risk factors of child mortality in Ethiopia: application of multilevel two-part model. PloS one. 2020;15(8):e0237640.
46. Oduse S, Zewotir T, North D. The impact of antenatal care on under-five mortality in Ethiopia: a difference-in-differences analysis. BMC pregnancy and childbirth. 2021;21:1-9.

Appendix

Table 2: Distribution and Association of covariate with Home Delivery and U5M

**Independent Variables with Categories**	**Frequency (%)**	**Home Delivery**		**U5M**	
		**Yes (%)**	**χ^2 − P value**	**Yes**	**χ^2 − P value**
**Age of Mother (in Year)**					
15-18	176(3.2)	82(.9)	0.0001	14(5.2)	
19-34	4065(73.7)	2026(71.3)		179(66.5)	
35-49	1146(23.1)	734(25.8)		76(28.3)	0.0000
**Religion of Mother**					
Orthodox	1857(33.7)	764(26.9)	0.0001	76(28.3)	
Catholic	17(0.3)	17(0.6)		0(0.0)	
Protestant	1458(26.4)	847(29.8)		48(17.8)	< 0.0001
Muslim	2095(38)	1142(40.2)		140(52.0)	
Others	90(1.6)	72(2.5)		5(1.9)	
**Place of residence**					
Urban	1362(24.7)	399(14.0)	0.0001	44(16.4)	< 0.0001
Rural	4156(75.3)	2443(86.0)		225(83.6)	
**Mother Educational Level**					
No Education	2960(58.5)	1955(68.8)	0.0000	158(58.5)	
Primary	1949(35.3)	810(28.5)		94(34.8)	
Secondary	415(7.5)	64(2.3)		14(5.2)	0.0001
Higher	194(3.5)	13(0.7)		4(1.5)	
**Region**					
Tigray	371(6.7)	98(3.4)	0.0001	14(5.2)	
Afar	86(1.6)	62(2.2)		5(1.8)	
Amhara	1047(19.0)	466(16.4)		44(16.2)	
Oromia	2206(40.0)	1297(45.0)		113(41.7)	<0.0001
Somali	408(7.4)	310(8.9)		41(15.1)	

Benishangul	67(1.2)	21(0.7)		5(1.8)	
SNNPR	1103(20.0)	576(20.3)		41(15.1)	
Gambela	24(0.4)	7(0.2)		2(0.7)	
Harari	17(0.3)	6(0.2)		1(0.4)	
Addis Ababa	156(2.8)	7(0.2)		3(1.1)	
Dire Dewa	30(0.5)	9(0.3)		2(0.7)	
**Wealth Index**					
Poor	2518(45.6)	1769(62.2)	0.0001	150(55.8)	
Middle	1041(18.9)	577(20.3)		47(17.5)	
Rich	1958(35.5)	496(17.5)		72(26.8)	< 0.0001
**Birth Interval of Child**					
High	1105(20)	733(25.8)	0.0001	96(35.7)	
Low	4413(80)	2109(74.2)		173(64.3)	< 0.0001
**No of Children Under Five in the House**					
High(>3)	4778(86.6)	2300(80.2)	0.0001	254(94.4)	
Low(<=3)	739(13.4)	543(19.8S)		15(5.6)	0.0001
**Sex of House Hold Head**					
Male	4331(78.5)	2191(77.1)	0.000	219(81.4)	0.0001
Female	1188(21.5)	652(22.9)		50(18.6)	
**Media Exposures**					
Yes	1882(34.1)	613(21.6)	0.0001	78(29.0)	0.0001
No	3635(65.9)	2229(78.4)		191(71.0)	
**During Pregnancy Counseling Health Worker**					
Yes	2074(37.6)	620(21.8)	0.0001	60(22.4)	0.0000
No	3443(62.4)	2222(78.2)		209(77.6)	
**Current Contraceptive Use**					
Yes	2242(40.6)	901(31.7)	0.0001	72(26.4)	0.0000
No	3275(59.4)	1941(68.3)		197(73.6)	
**Postnatal Check within 2 Months**					
Yes	535(9.7)	147(5.2)	0.0000	13(4.8)	0.000
No	4983(90.3)	2695(94.8)		257(95.2)	
**Number of Anti Natal Care (ANC) visit**					
aw Visit	1009(18.3)	863(30.8)	0.0001	65(24.3)	0.0004
No Visit	4509(50)	1979(69.2)		204(75.8)	
**Marital Status**					
Married	5184(94.0)	2669(93.9)	0.0000	250(92.9)	0.0000
Not	333(6.0)	173(6.1)		19(7.2)	
**Community level Anti Natal Visit**					
Low	3054(55.4)	2046(72.0)	0.0000	177(65.4)	0.0000
High	2463(44.6)	796(28.0)		92(34.6)	
**Community level Wealth Index**					
Low	2712(49.2)	1677(59.0)		154(57.0)	0.0000
High	2805(50.8)	1165(41.0)		116(43.0)	

**Community level Education**					
Low	3394(61.5)	1929(67.9)	0.0000	187(69.5)	0.0000
High	2124(38.5)	913(32.1)		82(30.5)	
**Community level Media Exposure**					
Low	2382(43.2)	1440(50.7)	0.0000	146(54.3)	0.0000
High	3135(56.8)	1402(49.3)		123(45.7)	

Table 3: Joint and marginal probability of Home Delivery and U5M.

**Responses**		**U5M**		**Marginal of Home Delivery**	**Odds Ratio (95% CI)**
		**No**	**Yes**		
**Home Delivery**	**No**	2688(51.2)	154(57.2)	2842(51.5)	2.19(1.89, 2.62)
	**Yes**	2560(48.8)	**115(42.8)**	2675(48.5)	
**Marginal of U5M**		3139(95.2)	269(4.8)	5517(1)	
**Measure of Association,** $\chi^2 = 3.83$, $P\_value = 0.0001$					

Table 4: Model Comparison for report among MI, MII, MIII and MIV and Variability between household levels

**Parameter**	**Model-I**	**Model-II**	**Model-III**	**Model-IV**
Variance	0.8967	0.8658	0.7791	0.4150
ICC	Ref			0.21
PCV(R^2)	Ref			0.54
AIC (smaller is better)	12381.06	7541.53	7524.07	7503.45
BIC (smaller is better)	12385.39	7681.86	7694.62	7591.28
Pearson Chi-Square /DF	0.94	1.00	0.91	0.94

Table 5: Parameter estimates of the multilevel bivariate modeling of Home Delivery and U5M.

Variables	Home Delivery		U5M	
	**Estimate (95% CI)**	**AOR**	**Estimate (95% CI)**	**AOR**
**Intercept**	-0.4113(-0.425, 0.9584)	0.662	-0.4113(-0.425, 0.9584)	0.662
**Mother's Age in year**				
15-18	0.53(0.15, 0.91)**	1.699	0.73(0.09, 1.37)**	2.075
19-34	Ref	1	Ref	1
35-49	0.17(0.01,0.34)*	1.185	0.30(0.03, 0.61)**	1.350
**Religion Respondents**				
Orthodox	0.53(0.16, 1.20)**	1.699	0.23(0.32, 1.47)*	1.258
Catholic	-2.91(--6.41, 0.59)	0.054	-1.85(-7.32, 3.62)	0.157
Protestant	0.07(-0.59, 0.72)	1.072	-0.03(-1.27, 1.22)	0.970
Muslim	1.06(0.39, 1.72)**	2.886	-0.03(-1.27, 1.22)	0.970
Others	Ref	1	Ref	1
**Place of Residence**				
Urban	Ref	1	Ref	1
Rural	0.46(0.24, 0.69)***	1.584	0.72(0.28, 1.17)***	2.054
**Mother Educational Level**				
No education	0.87(0.26, 1.48)***	2.386	0.25(0.17, 0.77)**	1.284
Primary	Ref	1	Ref	1
Secondary	-0.59(-0.92, -0.26)**	0.554	-0.13(-0.74, 0.49)	0.878
Higher	-0.81(-0.97, -0.65)**	0.445	-0.68(-1.85, -0.49)**	0.506
**Region**				
Tigray	3.11(0.97, 5.25)***	22.42	-0.16(-1.75, 1.43)	0.852
Afar	1.41(-0.81, 3.64)	4.096	0.17(-1.77, 2.10)	1.185
Amhara	2.37(0.24, 4.49)**	10.697	0.11(-1.39, 1.61)	1.116
Oromia	2.25(0.15, 4.34)**	9.488	-0.07(-1.47, 1.34)	0.932
Somalia	1.84(-0.31, 3.98)***	6.296	2.11(0.53, 3.69)***	8.248
Benishangul	3.14(0.90, 5.38)**	23.103	1.05(-0.88, 2.97)	2.857
SNNPR	2.74(0.63, 4.84)**	15.487	0.05(-1.44, 1.52	1.051
Gambela	3.32(0.90 5.74)**	27.660	0.77(-1.64, 3.19)	2.159
Harari	1.93(-0.63, 4.49)	6.889	0.49(-2.51, 3.48)	1.632

Addis Ababa	Ref	1	Ref	1
Dire Dewa	2.24(0.19, 4.65)***	9.393	0.81(-1.61, 3.23)**	2.247
**Wealth Index of Household**				
Poor	0.32(0.13, 0.51)**	1.377	0.17(0.12, 0.55)**	1.185
Middle	Ref	1	Ref	1
Rich	-1.27(-1.48, -1.06)*	0.281	-0.25(-0.67, -0.17)*	0.779
**Number of Under-five in Household**				
Low(<=3)	Ref	1	Ref	1
High(>3)	-0.54(-0.77, 0.33)	0.583	-1.82(-2.49, 1.23)	0.162
**Birth Interval of Child**				
Low	Ref	1	Ref	1
High	-0.06(-0.37, 0.25)	0.942	0.22(-0.44, 0.88)	1.246
**Sex of House Hold Head**				
Male	Ref	1	Ref	1
Female	0.02(-0.09, 0.53)	1.020	0.22(-0.19 ,0.42)	1.246
**Pregnancy Counseling**				
No	Ref	1	Ref	1
Yes	1.408(-0.654, 1.67)	4.088	-0.546(-0.145, 0.856)	0.579
**Media Exposures**				
No	Ref	1	Ref	1
Yes	-0.09(-0.28, -0.03)*	0.913	-0.07(-0.43, -0.27)*	0.932
**Marital Status**				
Not married	Ref	Ref	1	Ref
Married	0.18(-0.11, 0.48)	1.197	-0.07(-0.64, 0.49)	0.932
**Current Contraceptive Use**				
No	Ref	1	Ref	1
Yes	-0.24(-0.40, -0.09)**	0.786	-0.19(-0.52, -0.13)**	0.827
**Postnatal Check within 2 months**				
No	Ref	1	Ref	1
Yes	-0.48(-0.73, -0.23)**	0.619	-0.47(-1.08, -0.14)**	0.625
**Number ANC Visit**				
No Visit	Ref	1	Ref	1
Visit	-1.23(-1.45, -1.01)***	0.292	-0.24(-0.11, 0.58)	0.787
**Community Level Anti Natal Visit**				
Low	0.71(0.34, 1.08)**	2.034	0.45(0.22, 1.17)**	1.568
High	Ref	1	Ref	1
**Community Level Wealth Index**				
Low	0.15(0.02, 0.58)**	1.162	0.69(0.06, 1.39)**	1.993
High	Ref	1	Ref	
**Community Level Education**				
Low	0.14(0.02, 0.49)**	1.150	0.12(0.05, 0.72)**	1.127
High	Ref	1	Ref	1

**Community Level Media Exposure**				
Low	0.18(0.03, 4.31)**	1.197	0.08(0.06, 0.81)**	1.083
High	Ref	1	Ref	1

Measure of dependency; [OR (95% CI) =3.501 (1.267, 6.713)] ***.

* = significant at α = 0.05, ** = significant at α = 0.01 and *** = significant at α = 0.001.
