# Supplementary material for: Exploring the association between home delivery and under-five mortality in Ethiopia: A bivariate multilevel mixed-effects analysis
Source: PLoS One. 2026 Aug 12;21(8):e0355621. doi: 10.1371/journal.pone.0355621 (PMC13465812; doi:10.1371/journal.pone.0355621)
Supplement: Supplementary file 2 [file pone.0355621.s002.pdf]

Response to Reviewers and Editor

Manuscript ID: PONE-D-25-42341

Title: *Exploring the association between home delivery and under-five mortality in Ethiopia: A bivariate multilevel mixed-effects analysis*

Dear Dr. Alfredo Luis Fort,

We sincerely thank you for the opportunity to revise our manuscript and for your valuable comments and suggestions. We have carefully addressed all points raised and revised the manuscript accordingly. Below we summarize the major revisions and confirm compliance with the journal's requirements.

1. Language and Readability

As recommended, the manuscript has undergone comprehensive English editing by an academic language professional to improve clarity, coherence, and readability. Ambiguous or grammatically incorrect phrases were corrected throughout.

2. Editor's Comments (attached file)

We carefully addressed all editorial comments and corrections suggested in the annotated file. Each point raised was reviewed, and the corresponding section of the manuscript was revised. Key examples include:

- **Inconsistent statistics (55% vs 57%)**

  Editor comment: "Here you mention 55% but above you put '57%', it seems for the same statistics. Please review and use the correct one, and only once in this paragraph. Which is valid?"

  Author response: Thank you. The two figures originate from different sources-WHO (2022) and the UN Inter-agency Group for Child Mortality Estimation (2019). We

clarified this and revised the text to present each statistic once with clear source attribution:

"According to the 2022 WHO report, Sub-Saharan Africa accounted for 57% of global under-five deaths (≈2.8 million; 95% CI: 2.5–3.3 million)… A UN Inter-agency Group for Child Mortality Estimation analysis of 2019 data estimated that the region contributed 55% of global U5M…"

This removes duplication and ensures clarity regarding data sources.

Incorrect use of percentage for under-five mortality (U5M)

Editor comment: "'Also U5M was also higher in rural residents (83.6%).' This seems to be a mistake, since U5M is per thousand, not percentage. Please double-check."

Author response: Thank you for this observation. The 83.6% figure refers to the **proportion of surveyed mothers** who reported experiencing at least one child death before age five, not an under-five mortality *rate* per 1,000 live births. To clarify, U5M in our study was **categorized as a binary variable**, indicating whether a child died before reaching their fifth birthday (1 = died, 0 = survived). Accordingly, percentages reported in the Results section represent the proportion of mothers who experienced at least one under-five death.

3. Formatting and Style Requirements

The manuscript now fully follows **PLOS ONE's formatting guidelines**, including structure, references, and file naming conventions.

4. Ethics Statement

The ethics statement has been relocated to the **Methods section** only and removed from all other parts of the manuscript, as instructed.

5. Data Sharing

The dataset used in this study is publicly accessible from the **Demographic and Health Surveys (DHS) Program** website (https://dhsprogram.com/data/) upon free registration and approval of a data access request, as stated in the *Availability of data and materials* section of the manuscript.

6. References and Citations

All suggested references were reviewed, and relevant ones were incorporated to strengthen the Background and Discussion sections.

7. Figures and Supporting Files

All figure files were checked and verified using the **PACE** (Preflight Analysis and Conversion Engine) tool to ensure compliance with PLOS ONE's figure requirements.

8. Files Included in This Resubmission

1. **Response to Reviewers** (this document)
2. **Revised Manuscript with Track Changes**
3. **Clean Revised Manuscript (without Track Changes)**
4. **PACE-verified figure**

We believe these revisions have substantially improved the manuscript and addressed all concerns. We are sincerely grateful for your time, feedback, and consideration.

With kind regards,

Maru Zewdu Kassie

(on behalf of all co-authors)

Assosa University, Assosa, Ethiopia

Email: maruzewdu68@gmail.com
