# Supplementary material for: Exploring the association between home delivery and under-five mortality in Ethiopia: A bivariate multilevel mixed-effects analysis
Source: PLoS One. 2026 Aug 12;21(8):e0355621. doi: 10.1371/journal.pone.0355621 (PMC13465812; doi:10.1371/journal.pone.0355621)
Supplement: Supplementary file 3 [file pone.0355621.s004.pdf]

Date: **March 6, 2026**

To: **Editor-in-Chief**

PLOS ONE

Dear Editor-in-Chief,

Re: Manuscript Submission - [Exploring the association between home delivery and under-five mortality in Ethiopia: A bivariate multilevel mixed-effects analysis], with manuscript ID: PONE-D-25-42341R1.

We would like to express our gratitude to the reviewers and editor for their valuable comments and suggestions on our manuscript entitled "Exploring the association between home delivery and under-five mortality in Ethiopia: A bivariate multilevel mixed-effects analysis". We appreciate the time and effort invested in reviewing our work. In response to their comments, we have made significant revisions to address each point raised. Below, we provide a point-by-point response to the reviewers' comments. Our responses are prefaced by "Author Response" and are shown in blue to distinguish them from the reviewers' comments.

We believe that these revisions have significantly strengthened our manuscript and have addressed all the concerns raised by the reviewers. We hope that our revised work meets the standards of PLOS ONE and would be suitable for publication. We appreciate your consideration of our revised manuscript.

Thank you once again for your time and attention to our submission.

Sincerely,

Wondaya Fenta Zewdia &

Maru Zewdu Kassie,

Corresponding Authors

<u>In response to comments from Reviewer # 2</u>

Reviewer #2: Summary

This manuscript investigates the association between home delivery and under-five mortality in Ethiopia using data from the 2019 Ethiopian Mini Demographic and Health Survey. The topic is highly relevant to maternal and child health in low- and middle-income countries, and the use of nationally representative data is a clear strength. The application of a bivariate multilevel mixed-effects model to jointly analyze two correlated outcomes is methodologically interesting and potentially valuable.

However, several major issues require careful attention before the manuscript can be considered for publication. These relate mainly to causal interpretation, outcome definition, statistical clarity, internal consistency of results, and over interpretation of findings. Addressing these concerns will substantially improve the scientific rigor and clarity of the manuscript.

Author's Response: We sincerely thank the reviewer for the insightful and constructive comments. We have carefully considered all suggestions and have thoroughly revised and refined the manuscript to improve its scientific rigor, clarity, and consistency.

Major Comments

1. **Causal interpretation and language**: The manuscript uses causal language despite employing a cross-sectional design. Such analyses can identify associations but cannot establish causality or temporal order, especially for under-five mortality. The Results, Discussion, and Conclusions should consistently use associative language, describing home delivery as associated with higher odds of under-five mortality rather than as a causal determinant.

Author response: We thank the reviewer for this important comment. We have addressed this concern in the revised manuscript by removing causal language and consistently using associative terminology throughout the Results, Discussion, and Conclusions, in line with the cross-sectional study design.

2. **Definition and interpretation of under-five mortality:** Under-five mortality is defined as a binary indicator of whether a woman experienced at least one child death before age five, rather than the standard rate per 1,000 live births. Reported percentages represent proportions of mothers reporting child loss (unit of analysis: mother), not mortality rates. This distinction should be clearly stated in the Methods, Results, and Discussion sections.

Author response: We appreciate the reviewer's important clarification regarding the definition and interpretation of under-five mortality. In the revised manuscript, we have explicitly defined the outcome variable (under five mortality) in the Methods section (Variables in the Study, page 5, lines 180-183). Under-five mortality is clearly operationalized as a mother-level binary variable indicating whether a woman experienced at least one live-born child death before age five. We also explicitly state that this measure does not represent the conventional under-five mortality rate (deaths per 1,000 live births), but rather the proportion of mothers who experienced at least one under-five child death. Furthermore, we have ensured consistent wording throughout the Methods, Results, and Discussion sections to clarify that the unit of analysis is the mother and that all reported percentages refer to proportions of mothers, not child mortality rates.

3. **Statistical modeling clarity and transparency**: The statistical methods section contains extensive mathematical notation, but the conceptual explanation of the bivariate multilevel mixed-effects model is limited.

   Author response: We have substantially revised the Statistical Analysis section to provide a clearer conceptual explanation of the bivariate multilevel mixed-effects model.

4. **Internal consistency and interpretation of results:** The manuscript shows inconsistencies, such as unusually small chi-square statistics, counterintuitive antenatal care findings, and misinterpretation of model intercepts. Statistical outputs and interpretations should be reviewed for consistency and aligned with proper statistical and epidemiological meaning.

Author response: We have carefully reviewed all statistical outputs and revised the manuscript to ensure internal consistency. Counterintuitive findings related to antenatal care have been clarified, and the interpretation of model intercepts has been corrected to reflect their proper statistical meaning.

5. **Magnitude and stability of regional effects**: Some regional adjusted odds ratios are extremely large, with values exceeding twenty in certain regions. These estimates raise concerns about sparse data, model instability, or over-fitting. The manuscript does not provide sufficient information on regional sample sizes or event counts to assess the robustness of these findings. The authors should report sample sizes by region, discuss the possibility of sparse-data bias, and interpret large regional estimates with appropriate caution.

Author response: We appreciate the reviewer's important comment regarding the magnitude and stability of regional effects. The sample sizes and corresponding event distributions by region are provided in **Table 3 (Appendix section, page 22-23, lines 607-609)**, titled *"Distribution and Association of Covariates with Home Delivery and Under-Five Mortality."* We have now explicitly referenced this table in the main text to improve transparency regarding regional sample sizes and event counts. We have corrected this in the revised manuscript and acknowledge the substantial regional differences across Ethiopia. Several factors may contribute to this variability, including cultural differences, infrastructure disparities, instability, and religious diversity. In addition, we now discuss the potential for sparse-data bias in regions with relatively small sample sizes (e.g., Gambela, Harari, and Dire Dawa), and we interpret large adjusted odds ratios with appropriate caution, acknowledging the possibility of model instability due to limited event counts in some areas.

Minor Comments

6. **Organization and redundancy**: The Results section includes substantial interpretive content that overlaps with the Discussion. Reducing repetition and moving interpretation to the Discussion would improve clarity and structure.

Author response: We have restructured the manuscript to improve clarity and organization. The Results section now presents only objective statistical findings, while all interpretation and contextualization have been moved exclusively to the Discussion section. In addition, redundant content has been carefully removed to enhance conciseness and readability.

7. **Language and readability**: Although the manuscript has undergone language editing, minor grammatical issues and repetitive phrasing remain. Additional polishing would improve readability.

Author response: We have revised the manuscript and conducted additional careful language editing to correct minor grammatical issues and eliminate repetitive phrasing, thereby improving overall clarity and readability.

8. **Figures and tables**: Figure 1 provides limited information beyond what is already presented in the tables and may be redundant. The authors may wish to reconsider its inclusion.

Author response: Thank you for this thoughtful suggestion. We respectfully believe that Figure 1 is important for the manuscript, as it visually presents the distribution and association between the two outcome variables in a clear and intuitive manner. While related information appears in the tables, the figure enhances understanding by providing a graphical summary of the relationship. Therefore, we have retained Figure 1 in the revised manuscript.

9. **Terminology consistency**: Terminology related to under-five mortality should be used consistently throughout the manuscript to avoid ambiguity.
   Author response: Thank you for the comment. We have standardized the terminology for under-five mortality throughout the manuscript to ensure clarity and consistency.

Reviewer Comment - Overall Recommendation: Major revision

Comment: The study addresses an important public health question and uses a potentially valuable analytical approach. However, substantial revisions are required to improve conceptual clarity, statistical transparency, consistency of interpretation, and alignment between the study design and the conclusions.

Author response: We appreciate the reviewer's constructive feedback. We have carefully revised the manuscript to enhance conceptual clarity, improve statistical transparency, ensure consistent interpretation, and better align the study design with the conclusions.

Author response for the attached comments

Reviewer Comment: This manuscript addresses an important public health issue in Ethiopia and applies an interesting bivariate multilevel mixed-effects approach. However, significant methodological and reporting gaps remain.

Author Response: We sincerely thank the reviewer for recognizing the importance of our study and for the constructive comments. We have carefully addressed each point.

1. Could the authors clarify the sampling procedure, including the total number of participants invited, the response rate, recruitment method, inclusion/exclusion criteria, and how non-responses or incomplete cases were handled?

Author response: We have addressed the reviewer's comment regarding the sampling procedure in the revised manuscript (page 5, lines 110–120).

2. Would the authors provide a flow diagram (as recommended by STROBE) showing how the final sample of 5,517 women was obtained?

Author response: We have incorporated a flow diagram showing the selection of the final sample of 5,517 women in the revised manuscript (page 6, lines 132–139).

3. How were missing values handled in the analysis?, Could the authors provide a table showing the proportion of missing data for each variable?

   Author response: Thank you for your comment. Missing values were handled using the multiple imputation method. Regarding the proportion of missing data for each variable, only variables such as antenatal care, media exposure, and maternal education had small amounts of missing values. Since the proportions were very low, they were handled using multiple imputation. A description of this procedure has been added to the revised manuscript.

4. Did the authors apply DHS sampling weights in the analysis?, If not, could they explain why, and if so, could they clarify exactly how the weights were incorporated into the multilevel model?

   Author response: Yes, the data for this study were obtained from the 2019 Ethiopian Mini Demographic and Health Survey (EMDHS). Individual sample weights (v005/1,000,000) were applied in all analyses to account for over- and under-sampling, and the weights were incorporated into the multilevel model accordingly.

5. Could the authors complete and submit a STROBE checklist to ensure compliance with reporting standards for observational studies?

Author response: We have completed the STROBE checklist and submitted it alongside the revised manuscript to ensure full compliance with reporting standards for observational studies.
