# Supplementary material for: Exploring the association between home delivery and under-five mortality in Ethiopia: A bivariate multilevel mixed-effects analysis
Source: PLoS One. 2026 Aug 12;21(8):e0355621. doi: 10.1371/journal.pone.0355621 (PMC13465812; doi:10.1371/journal.pone.0355621)
Supplement: Supplementary file 4 [file pone.0355621.s005.pdf]

Date: **July 10, 2026**

To: **Editor-in-Chief**

PLOS ONE

Dear Editor-in-Chief,

Re: Manuscript Submission - [Exploring the association between home delivery and under-five mortality in Ethiopia: A bivariate multilevel mixed-effects analysis], with manuscript ID: PONE-D-25-42341R2.

We would like to express our gratitude to the reviewers and editor for their valuable comments and suggestions on our manuscript entitled “Exploring the association between home delivery and under-five mortality in Ethiopia: A bivariate multilevel mixed-effects analysis”. We appreciate the time and effort invested in reviewing our work. In response to their comments, we have made significant revisions to address each point raised. Below, we provide a point-by-point response to the reviewers' comments. Our responses are prefaced by “Author Response” and are shown in blue to distinguish them from the reviewers’ comments.

We believe that these revisions have significantly strengthened our manuscript and have addressed all the concerns raised by the reviewers. We hope that our revised work meets the standards of PLOS ONE and would be suitable for publication. We appreciate your consideration of our revised manuscript.

Thank you once again for your time and attention to our submission.

Sincerely,

Wondaya Fenta Zewdia &

Maru Zewdu Kassie,

Corresponding Authors

In response to comments from Reviewer # 2

General Comment

Reviewer Comment: Thank you to the authors for the revisions. While several aspects of the manuscript have improved, important concerns remain insufficiently addressed, particularly regarding causal interpretation, statistical validity, and consistency of interpretation. These issues need further revision before the manuscript can be considered for publication.

Author Response: We sincerely thank the reviewer for the positive assessment and constructive recommendations. Following these suggestions, we thoroughly revised the manuscript to improve the consistency of the outcome definition, eliminate causal language, clarify the statistical methodology, simplify the interpretation of the bivariate multilevel model, and improve the presentation of the results. All requested revisions have been incorporated throughout the manuscript.

Major Comments

1. **Persistent causal language and over-interpretation**: Despite revisions, the manuscript continues to use causal language inconsistent with a cross-sectional design "implying causality". Statements such as:
   - "children born at home are more likely to die"
   - "protective effect of institutional delivery"
   - "43% of deaths were attributed to home delivery"

   imply causality or attribution, which cannot be supported by the study design. The manuscript must consistently use associative language and avoid any implication of causation or attribution.

   Author Response: We carefully reviewed the entire manuscript and removed all remaining causal or attributional language. Expressions such as *"children born at home are more likely to die," "protective effect of institutional delivery," "reduces mortality,"* and *"43% of deaths were attributed to home delivery"* have been removed or revised. Throughout the manuscript, we now consistently use association-based terminology such as *"was associated with," "had higher odds," "was linked to,"* and *"may be associated with."* We also explicitly state in the Results, Discussion, Conclusion, and Strengths and

Limitations sections that, because this study used a cross-sectional design, the findings represent statistical associations and should not be interpreted as causal relationships.

2. **Inconsistency between outcome definition and interpretation**: The outcome is defined as a mother-level binary variable (experience of at least one under-five death). However, results and interpretations are frequently presented at the child level (e.g., "children born at home…"). This inconsistency is conceptually incorrect and must be resolved throughout the manuscript.

   Author Response: We carefully revised the manuscript to ensure that the interpretation is fully consistent with the outcome analyzed. The Methods section now clearly defines under-five mortality as a mother-level binary variable indicating whether a woman experienced at least one under-five child death. We subsequently revised the Abstract, Results, Discussion, Conclusion, and Strengths and Limitations sections to consistently interpret the findings at the mother level. Statements referring to child-level mortality were replaced with wording such as *"maternal experience of under-five child death"* or *"mothers who reported at least one under-five child death,"* thereby ensuring conceptual consistency throughout the manuscript.

3. **Statistical inconsistency in chi-square results**: The reported chi-square statistic (χ^2 = 3.83) with $p < 0.0001$ appears implausible and suggests either a reporting or calculation error. All statistical results should be carefully verified and corrected.

   Author Response: We thank the reviewer for identifying this reporting error. The reported chi-square statistic (χ^2 = 3.83) was a typographical error. The correct value is **χ^2 = 14.83**, which is consistent with the reported **$p < 0.0001$**. We have verified all statistical results and corrected this value in the revised manuscript.
   Changes made: Corrected the chi-square statistic from **χ^2 = 3.83** to **χ^2 = 14.83** after verifying the analysis.

4. **Continued misinterpretation of model intercepts:** The intercept term is still interpreted substantively. Intercepts represent baseline log-odds under reference conditions and should not be given epidemiological interpretation.

   Author Response: We fully agree that the regression intercept does not have a meaningful epidemiological interpretation in this context. Accordingly, we removed the paragraph interpreting the intercept from the Results section. The revised Results now focus exclusively on the interpretation of the estimated regression coefficients and adjusted odds ratios for the explanatory variables.

5. **Limited improvement in statistical modeling clarity:** While the Methods section includes additional explanation, the model remains difficult to interpret. In particular:
   • the practical meaning of the association parameter is still unclear
   • the “loop rule” is referenced without sufficient explanation
   A more intuitive and applied explanation is needed.

   Author Response: We sincerely thank the reviewer for this valuable suggestion. In response, we substantially revised the Statistical Analysis section to improve the clarity and accessibility of the multilevel bivariate modeling approach. The explanation of the association parameter has been rewritten using simpler and more intuitive language. Specifically, we now explain that the association parameter, expressed as a log odds ratio, quantifies the adjusted relationship between home delivery and maternal experience of under-five child death after accounting for individual- and community-level characteristics and community-level clustering. We also clarify that an odds ratio greater than one indicates a positive association, an odds ratio equal to one indicates no association, and an odds ratio less than one indicates a negative association. In addition, we removed the reference to the “loop rule” because it was unnecessary for interpreting the model and could confuse readers. Finally, we expanded the rationale for using the bivariate multilevel mixed-effects logistic regression model and explained how it jointly models two correlated binary outcomes while accounting for the hierarchical structure of the data.

6. **Large regional effects remain insufficiently justified:** Although sample sizes are now provided, extremely large adjusted odds ratios (e.g., >20) remain concerning. The manuscript should:
    - discuss potential model instability more explicitly
    - consider sensitivity analyses or model diagnostics
    - interpret these estimates with greater caution

    Author Response: We appreciate the reviewer for this important comment. We have revised the Discussion to provide a more cautious interpretation of the regional estimates. Specifically, we now acknowledge that the very large adjusted odds ratios observed for some regions may partly reflect relatively small regional sample sizes, sparse data, or residual heterogeneity, which can result in unstable parameter estimates. We therefore advise readers to interpret these estimates cautiously. In addition, we re-examined the fitted model using standard diagnostic procedures and confirmed that the final model satisfied the convergence criteria and demonstrated an adequate overall fit based on model selection criteria. We have also noted this issue as a limitation of the study and recommend that future studies with larger regional samples further investigate these regional differences.

Minor Comments

Reviewer Comment: Some improvements in structure are noted, but minor language issues remain.

Author Response: We sincerely thank the reviewer for this suggestion. We carefully proofread the entire manuscript and revised numerous sentences to improve grammar, readability, and consistency. Redundant wording was removed, sentence structure was improved, and the overall flow of the manuscript was enhanced while preserving the original scientific content.

Reviewer Comment: Terminology should be consistently aligned with the unit of analysis (mother vs child).

Author Response: We thoroughly reviewed the manuscript and ensured that the terminology is consistently aligned with the mother-level outcome analyzed in this study. Child-level

interpretations were revised throughout the manuscript to accurately reflect maternal experience of under-five child death.

Reviewer Comment: Thank you for incorporating the participant flow. It can be cosmetically improved by aligning the arrows. It should also show the ones that were excluded to be complete.

Author Response: The participant flow diagram has been revised to improve its visual presentation by aligning the arrows and enhancing the overall layout. We also updated the figure to explicitly indicate the number of participants excluded at each stage of the selection process, together with the reasons for exclusion, thereby providing a more complete and transparent description of participant selection.

Response to Reviewer #3

General Comment: The manuscript investigates an important public health issue using nationally representative Ethiopian DHS data and applies a useful bivariate multilevel modeling framework. The revised version has improved in terms of organization, terminology consistency, and reduction of causal language. The study has potential scientific value and contributes to understanding the association between home delivery and maternal experience of under-five mortality in Ethiopia. Some concerns still remain unaddressed.

Author Response: We sincerely thank the reviewer for the thoughtful evaluation of our manuscript and for recognizing its public health relevance, the use of nationally representative Ethiopian Mini DHS data, and the application of a bivariate multilevel modelling framework. We greatly appreciate the constructive comments and have carefully revised the manuscript to address all remaining concerns. Our detailed responses are presented below.

1. Some interpretations continue to imply causality despite the cross-sectional design, particularly in the Discussion and Conclusion sections.

Author Response: We sincerely thank the reviewer for this important observation. We carefully reviewed the entire manuscript and removed all remaining causal language. Expressions implying causality, such as *"protective effect," "reduces mortality," "children born at home are more likely to die,"* and *"attributed to"* have been replaced with appropriate association-based terminology, including *"was associated with," "had higher odds," "was linked to,"* and *"may*

be associated with." In addition, we explicitly state in the Discussion, Conclusion, and Strengths and Limitations sections that the cross-sectional study design identifies statistical associations but does not permit causal inference.

2. There are inconsistencies in the interpretation of ANC findings and the operational definition of under-five mortality, where mother-level outcomes are occasionally discussed as child-level mortality rates.

Author Response: We appreciate the reviewer for identifying this important issue. We carefully revised the interpretation of the antenatal care (ANC) findings to ensure that it is fully consistent with the model estimates and the reference category used in the analysis. Furthermore, we thoroughly reviewed the manuscript to ensure that the operational definition of under-five mortality is consistently presented as a mother-level binary outcome indicating whether a woman reported experiencing at least one under-five child death. All child-level interpretations were revised accordingly in the Abstract, Methods, Results, Discussion, and Conclusion to maintain consistency with the unit of analysis.

3. Regional odds ratios appear extremely large that suggest possible sparse-data bias or model instability, and these findings require more cautious interpretation.

Author Response: We sincerely thank the reviewer for this valuable suggestion. We have revised the Discussion to provide a more cautious interpretation of the regional estimates. Specifically, we now acknowledge that the large adjusted odds ratios observed for some regions may partly reflect relatively small sample sizes, sparse data, or residual heterogeneity, which can produce unstable parameter estimates. We therefore advise readers to interpret these findings cautiously. We also re-examined the fitted model using standard diagnostic procedures and confirmed that the final model achieved satisfactory convergence and model fit. This limitation has also been acknowledged in the manuscript, and we recommend that future studies with larger regional samples further investigate these regional differences.

4. The statistical methods section still needs simplification and clearer explanation of the bivariate multilevel framework.

Author Response: In response, we substantially revised the Statistical Analysis section to improve its clarity and accessibility. We expanded the rationale for using the bivariate multilevel mixed-effects logistic regression model and explained, in simpler language, that it simultaneously models two correlated binary outcomes while accounting for the hierarchical structure of the data. We also simplified the description of the association parameter by explaining its practical interpretation as the adjusted relationship between home delivery and maternal experience of under-five child death after accounting for individual- and community-level covariates and clustering. In addition, we removed the reference to the "loop rule" because it was unnecessary and could confuse readers.

5. Minor grammatical issues and repetitive statements are also present throughout the manuscript.

Author Response: We sincerely thank the reviewer for this helpful suggestion. We carefully proofread the entire manuscript and revised numerous sentences to improve grammar, readability, and consistency. Repetitive statements were removed, sentence structure was improved, and terminology was standardized throughout the manuscript while preserving the original scientific content.

6. The study is relevant and methodologically interesting, but moderate revision is still required to improve statistical clarity, interpretation consistency, and overall readability before publication consideration.

Author Response: We sincerely appreciate the reviewer's positive evaluation of the scientific relevance and methodological contribution of our study. Following the reviewer's recommendations, we comprehensively revised the manuscript to improve statistical clarity, strengthen consistency in the interpretation of the findings, simplify the presentation of the bivariate multilevel modelling framework, eliminate remaining causal language, and improve the overall readability of the manuscript. We believe that these revisions have substantially strengthened the manuscript and addressed all of the reviewer's concerns.

Thank You!!!
