# Supplementary material for: Exploring the association between home delivery and under-five mortality in Ethiopia: A bivariate multilevel mixed-effects analysis
Source: PLoS One. 2026 Aug 12;21(8):e0355621. doi: 10.1371/journal.pone.0355621 (PMC13465812; doi:10.1371/journal.pone.0355621)
Supplement: Supplementary file 5 [file pone.0355621.s003.docx]

This manuscript addresses an important public health issue in Ethiopia and applies an interesting bivariate multilevel mixed-effects approach. However, significant methodological and reporting gaps remain

1. Could the authors clarify the sampling procedure, including the total number of participants invited, the response rate, recruitment method, inclusion/exclusion criteria, and how non-responses or incomplete cases were handled?
2. Would the authors provide a flow diagram (as recommended by STROBE) showing how the final sample of 5,517 women was obtained?
3. How were missing values handled in the analysis?, Could the authors provide a table showing the proportion of missing data for each variable?
4. Did the authors apply DHS sampling weights in the analysis?, If not, could they explain why, and if so, could they clarify exactly how the weights were incorporated into the multilevel model?
5. Could the authors complete and submit a STROBE checklist to ensure compliance with reporting standards for observational studies?
