# Supplementary material for: Exploring the association between home delivery and under-five mortality in Ethiopia: A bivariate multilevel mixed-effects analysis
Source: PLoS One. 2026 Aug 12;21(8):e0355621. doi: 10.1371/journal.pone.0355621 (PMC13465812; doi:10.1371/journal.pone.0355621)
Supplement: Peer review history

# Editor report: Decision Letter 0

**DOI:** 10.1371/journal.pone.0355621.r001

**Authors:** Alfredo Luis Fort

8 Sep 2025

PONE-D-25-42341

Exploring the association between home delivery and under-five mortality in Ethiopia: A bivariate multilevel mixed-effects analysis

PLOS ONE

Dear Dr. Kassie,

Thank you for submitting your manuscript to PLOS ONE. After careful consideration, we feel that it has merit but does not fully meet PLOS ONE’s publication criteria as it currently stands. Therefore, we invite you to submit a revised version of the manuscript that addresses the points raised during the review process.

Please submit your revised manuscript by Oct 23 2025 11:59PM. If you will need more time than this to complete your revisions, please reply to this message or contact the journal office at plosone@plos.org. When you're ready to submit your revision, log on to https://www.editorialmanager.com/pone/ and select the 'Submissions Needing Revision' folder to locate your manuscript file.

Please include the following items when submitting your revised manuscript:

- A rebuttal letter that responds to each point raised by the academic editor and reviewer(s). You should upload this letter as a separate file labeled 'Response to Reviewers'.
- A marked-up copy of your manuscript that highlights changes made to the original version. You should upload this as a separate file labeled 'Revised Manuscript with Track Changes'.
- An unmarked version of your revised paper without tracked changes. You should upload this as a separate file labeled 'Manuscript'.

If you would like to make changes to your financial disclosure, please include your updated statement in your cover letter. Guidelines for resubmitting your figure files are available below the reviewer comments at the end of this letter.

If applicable, we recommend that you deposit your laboratory protocols in protocols.io to enhance the reproducibility of your results. Protocols.io assigns your protocol its own identifier (DOI) so that it can be cited independently in the future. For instructions see: https://journals.plos.org/plosone/s/submission-guidelines#loc-laboratory-protocols. Additionally, PLOS ONE offers an option for publishing peer-reviewed Lab Protocol articles, which describe protocols hosted on protocols.io. Read more information on sharing protocols at https://plos.org/protocols?utm_medium=editorial-email&utm_source=authorletters&utm_campaign=protocols.

We look forward to receiving your revised manuscript.

Kind regards,

Alfredo Luis Fort, M.D., M.Sc., Ph.D.

Academic Editor

PLOS ONE

Journal Requirements:

When submitting your revision, we need you to address these additional requirements.

1. Please ensure that your manuscript meets PLOS ONE's style requirements, including those for file naming. The PLOS ONE style templates can be found at https://journals.plos.org/plosone/s/file?id=wjVg/PLOSOne_formatting_sample_main_body.pdf and https://journals.plos.org/plosone/s/file?id=ba62/PLOSOne_formatting_sample_title_authors_affiliations.pdf

2. Please note that PLOS One has specific guidelines on code sharing for submissions in which author-generated code underpins the findings in the manuscript. In these cases, we expect all author-generated code to be made available without restrictions upon publication of the work. Please review our guidelines at https://journals.plos.org/plosone/s/materials-and-software-sharing#loc-sharing-code and ensure that your code is shared in a way that follows best practice and facilitates reproducibility and reuse.

3. Your ethics statement should only appear in the Methods section of your manuscript. If your ethics statement is written in any section besides the Methods, please move it to the Methods section and delete it from any other section. Please ensure that your ethics statement is included in your manuscript, as the ethics statement entered into the online submission form will not be published alongside your manuscript.

4. If the reviewer comments include a recommendation to cite specific previously published works, please review and evaluate these publications to determine whether they are relevant and should be cited. There is no requirement to cite these works unless the editor has indicated otherwise.

Additional Editor Comments :

I was about to invite reviewers but when looking at the manuscript, there were several errors and lack of clarity in the construction of phrases, that I have decided to send it back to you, the authors, to fully make it readable and have in checked by someone fully knowledgeable in English language. Please look in detail to all the comments and suggestions I have made in the attached file, and correct all the misinformation plus have it checked by someone fully skilled in written English academic language, before re-submitting. Thanks.

[Note: HTML markup is below. Please do not edit.]

[NOTE: If reviewer comments were submitted as an attachment file, they will be attached to this email and accessible via the submission site. Please log into your account, locate the manuscript record, and check for the action link "View Attachments". If this link does not appear, there are no attachment files.]

While revising your submission, please upload your figure files to the Preflight Analysis and Conversion Engine (PACE) digital diagnostic tool, https://pacev2.apexcovantage.com/. PACE helps ensure that figures meet PLOS requirements. To use PACE, you must first register as a user. Registration is free. Then, login and navigate to the UPLOAD tab, where you will find detailed instructions on how to use the tool. If you encounter any issues or have any questions when using PACE, please email PLOS at figures@plos.org. Please note that Supporting Information files do not need this step.

Attachment Submitted filename: PONE-D-25-42341-AF.pdf

# Author comment: Author response to Decision Letter 1

**DOI:** 10.1371/journal.pone.0355621.r002

23 Oct 2025

Response to Reviewers and Editor

Manuscript ID: PONE-D-25-42341

Title: Exploring the association between home delivery and under-five mortality in Ethiopia: A bivariate multilevel mixed-effects analysis

Dear Dr. Alfredo Luis Fort,

We sincerely thank you for the opportunity to revise our manuscript and for your valuable comments and suggestions. We have carefully addressed all points raised and revised the manuscript accordingly. Below we summarize the major revisions and confirm compliance with the journal’s requirements.

1. Language and Readability

As recommended, the manuscript has undergone comprehensive English editing by an academic language professional to improve clarity, coherence, and readability. Ambiguous or grammatically incorrect phrases were corrected throughout.

2. Editor’s Comments (attached file)

We carefully addressed all editorial comments and corrections suggested in the annotated file. Each point raised was reviewed, and the corresponding section of the manuscript was revised. Key examples include:

• Inconsistent statistics (55% vs 57%)

Editor comment: “Here you mention 55% but above you put ‘57%’, it seems for the same statistics. Please review and use the correct one, and only once in this paragraph. Which is valid?”

Author response: Thank you. The two figures originate from different sources-WHO (2022) and the UN Inter-agency Group for Child Mortality Estimation (2019). We clarified this and revised the text to present each statistic once with clear source attribution:

“According to the 2022 WHO report, Sub-Saharan Africa accounted for 57% of global under-five deaths (≈2.8 million; 95% CI: 2.5–3.3 million)… A UN Inter-agency Group for Child Mortality Estimation analysis of 2019 data estimated that the region contributed 55% of global U5M…”

This removes duplication and ensures clarity regarding data sources.

Incorrect use of percentage for under-five mortality (U5M)

Editor comment: “‘Also U5M was also higher in rural residents (83.6%).’ This seems to be a mistake, since U5M is per thousand, not percentage. Please double-check.”

Author response: Thank you for this observation. The 83.6% figure refers to the proportion of surveyed mothers who reported experiencing at least one child death before age five, not an under-five mortality rate per 1,000 live births. To clarify, U5M in our study was categorized as a binary variable, indicating whether a child died before reaching their fifth birthday (1 = died, 0 = survived). Accordingly, percentages reported in the Results section represent the proportion of mothers who experienced at least one under-five death.

3. Formatting and Style Requirements

The manuscript now fully follows PLOS ONE’s formatting guidelines, including structure, references, and file naming conventions.

4. Ethics Statement

The ethics statement has been relocated to the Methods section only and removed from all other parts of the manuscript, as instructed.

5. Data Sharing

The dataset used in this study is publicly accessible from the Demographic and Health Surveys (DHS) Program website (https://dhsprogram.com/data/) upon free registration and approval of a data access request, as stated in the Availability of data and materials section of the manuscript.

6. References and Citations

All suggested references were reviewed, and relevant ones were incorporated to strengthen the Background and Discussion sections.

7. Figures and Supporting Files

All figure files were checked and verified using the PACE (Preflight Analysis and Conversion Engine) tool to ensure compliance with PLOS ONE’s figure requirements.

8. Files Included in This Resubmission

1. Response to Reviewers (this document)

2. Revised Manuscript with Track Changes

3. Clean Revised Manuscript (without Track Changes)

4. PACE-verified figure

We believe these revisions have substantially improved the manuscript and addressed all concerns. We are sincerely grateful for your time, feedback, and consideration.

With kind regards,

Maru Zewdu Kassie

(on behalf of all co-authors)

Assosa University, Assosa, Ethiopia

Email: maruzewdu68@gmail.com

Attachment Submitted filename: Author Response.pdf

# Peer review: Decision Letter 1

**DOI:** 10.1371/journal.pone.0355621.r003

**Authors:** Emmanuel O Adewuyi

16 Jan 2026

PONE-D-25-42341R1

Exploring the association between home delivery and under-five mortality in Ethiopia: A bivariate multilevel mixed-effects analysis

PLOS One

Dear Dr. Kassie,

Thank you for submitting your manuscript to PLOS ONE. After careful consideration, we feel that it has merit but does not fully meet PLOS ONE’s publication criteria as it currently stands. Therefore, we invite you to submit a revised version of the manuscript that addresses the points raised during the review process.

==============================

**ACADEMIC EDITOR:**

Reviewers have raised some major issues in this manuscript. Comments related to methods are critical and need to be carefully and satisfactorily addressed. Authors need to explain why they used a different definition for under-five mortality and not following the conventional approach to this type of study. Issues around over-stating results and internal consistency need to be carefully addressed.

==============================

Please submit your revised manuscript by Mar 02 2026 11:59PM. If you will need more time than this to complete your revisions, please reply to this message or contact the journal office at plosone@plos.org. When you're ready to submit your revision, log on to https://www.editorialmanager.com/pone/ and select the 'Submissions Needing Revision' folder to locate your manuscript file.

Please include the following items when submitting your revised manuscript:

- A letter that responds to each point raised by the academic editor and reviewer(s). You should upload this letter as a separate file labeled 'Response to Reviewers'.
- A marked-up copy of your manuscript that highlights changes made to the original version. You should upload this as a separate file labeled 'Revised Manuscript with Track Changes'.
- An unmarked version of your revised paper without tracked changes. You should upload this as a separate file labeled 'Manuscript'.

If you would like to make changes to your financial disclosure, please include your updated statement in your cover letter. Guidelines for resubmitting your figure files are available below the reviewer comments at the end of this letter.

If applicable, we recommend that you deposit your laboratory protocols in protocols.io to enhance the reproducibility of your results. Protocols.io assigns your protocol its own identifier (DOI) so that it can be cited independently in the future. For instructions see: https://journals.plos.org/plosone/s/submission-guidelines#loc-laboratory-protocols. Additionally, PLOS ONE offers an option for publishing peer-reviewed Lab Protocol articles, which describe protocols hosted on protocols.io. Read more information on sharing protocols at https://plos.org/protocols?utm_medium=editorial-email&utm_source=authorletters&utm_campaign=protocols.

We look forward to receiving your revised manuscript.

Kind regards,

Emmanuel O Adewuyi, BPharm, MPH, PhD

Academic Editor

PLOS One

Journal Requirements:

If the reviewer comments include a recommendation to cite specific previously published works, please review and evaluate these publications to determine whether they are relevant and should be cited. There is no requirement to cite these works unless the editor has indicated otherwise.

[Note: HTML markup is below. Please do not edit.]

Reviewers' comments:

Reviewer's Responses to Questions

**Comments to the Author**

1. If the authors have adequately addressed your comments raised in a previous round of review and you feel that this manuscript is now acceptable for publication, you may indicate that here to bypass the “Comments to the Author” section, enter your conflict of interest statement in the “Confidential to Editor” section, and submit your "Accept" recommendation.

Reviewer #1: All comments have been addressed

Reviewer #2: (No Response)

**********

2. Is the manuscript technically sound, and do the data support the conclusions?

The manuscript must describe a technically sound piece of scientific research with data that supports the conclusions. Experiments must have been conducted rigorously, with appropriate controls, replication, and sample sizes. The conclusions must be drawn appropriately based on the data presented.

Reviewer #1: Yes

Reviewer #2: Yes

**********

3. Has the statistical analysis been performed appropriately and rigorously?

Reviewer #1: N/A

Reviewer #2: No

**********

4. Have the authors made all data underlying the findings in their manuscript fully available?

The PLOS Data policy requires authors to make all data underlying the findings described in their manuscript fully available without restriction, with rare exception (please refer to the Data Availability Statement in the manuscript PDF file). The data should be provided as part of the manuscript or its supporting information, or deposited to a public repository. For example, in addition to summary statistics, the data points behind means, medians and variance measures should be available. If there are restrictions on publicly sharing data—e.g. participant privacy or use of data from a third party—those must be specified.

Reviewer #1: No

Reviewer #2: Yes

**********

5. Is the manuscript presented in an intelligible fashion and written in standard English?

PLOS ONE does not copyedit accepted manuscripts, so the language in submitted articles must be clear, correct, and unambiguous. Any typographical or grammatical errors should be corrected at revision, so please note any specific errors here.

Reviewer #1: No

Reviewer #2: Yes

**********

6. Review Comments to the Author

Please use the space provided to explain your answers to the questions above. You may also include additional comments for the author, including concerns about dual publication, research ethics, or publication ethics. (Please upload your review as an attachment if it exceeds 20,000 characters)

Reviewer #1: (No Response)

Reviewer #2: Summary

This manuscript investigates the association between home delivery and under-five mortality in Ethiopia using data from the 2019 Ethiopian Mini Demographic and Health Survey. The topic is highly relevant to maternal and child health in low- and middle-income countries, and the use of nationally representative data is a clear strength. The application of a bivariate multilevel mixed-effects model to jointly analyze two correlated outcomes is methodologically interesting and potentially valuable.

However, several major issues require careful attention before the manuscript can be considered for publication. These relate mainly to causal interpretation, outcome definition, statistical clarity, internal consistency of results, and overinterpretation of findings. Addressing these concerns will substantially improve the scientific rigor and clarity of the manuscript.

Major Comments

1. Causal interpretation and language

Although the study uses a cross-sectional design, the manuscript frequently uses language that implies causality. Statements suggesting that home delivery increases under-five mortality, that home delivery is a significant risk factor, or that a specific proportion of under-five deaths is attributable to home delivery are not supported by the study design. Cross-sectional analyses can identify associations but cannot establish temporal order or causality, particularly for an outcome such as under-five mortality, which is influenced by many postnatal and contextual factors.

The conclusions and several sections of the Results and Discussion should be revised to consistently use associative language. Home delivery should be described as being associated with higher odds of under-five mortality rather than as a causal determinant.

2. Definition and interpretation of under-five mortality

Under-five mortality in this study is defined as a binary indicator reflecting whether a woman experienced at least one child death before age five. This differs from the standard definition of under-five mortality as deaths per 1,000 live births. While this operationalization may be analytically appropriate, it is not clearly or consistently communicated throughout the manuscript.

In several places, percentages are reported in a way that may be misinterpreted as mortality rates. For example, statements indicating that under-five mortality was higher in rural residents expressed as percentages represent proportions of mothers reporting at least one child death rather than mortality rates.

The manuscript should clearly emphasize that the unit of analysis is the mother rather than the individual child, that under-five mortality reflects maternal experience of child loss, and that reported percentages are proportions rather than rates. This clarification should be made explicit in the Methods, Results, and Discussion sections.

3. Statistical modeling clarity and transparency

The statistical methods section contains extensive mathematical notation, but the conceptual explanation of the bivariate multilevel mixed-effects model is limited. It is not sufficiently clear how the joint model accounts for correlation between home delivery and under-five mortality, how the association parameter is interpreted in practical terms, or how random effects are specified across outcomes.

The Results section also refers to concepts such as the loop rule without sufficient explanation in the Methods. In addition, the division of analytical tasks between STATA and R is not clearly described, which limits reproducibility.

The manuscript would benefit from a clearer and more intuitive explanation of the modeling approach, a concise interpretation of the joint association parameter, and explicit information on which software and procedures were used for each analytical step.

4. Internal consistency and interpretation of results

Several internal inconsistencies were noted across the manuscript. The reported chi-square statistics appear small relative to the extremely small p-value reported. Some findings related to antenatal care appear counterintuitive, particularly where lack of antenatal care is described as being associated with lower odds of home delivery, and these results are not adequately explained.

In addition, intercepting terms from the multilevel model are interpreted substantively in a way that is not statistically appropriate. Intercepts represent baseline log odds under reference conditions and should not be interpreted as meaningful population-level effects.

All statistical outputs should be carefully reviewed for consistency across tables and text, and interpretations should be revised to align with statistical meaning and epidemiological plausibility.

5. Magnitude and stability of regional effects

Some regional adjusted odds ratios are extremely large, with values exceeding twenty in certain regions. These estimates raise concerns about sparse data, model instability, or overfitting. The manuscript does not provide sufficient information on regional sample sizes or event counts to assess the robustness of these findings.

The authors should report sample sizes by region, discuss the possibility of sparse-data bias, and interpret large regional estimates with appropriate caution.

Minor Comments

6. Organization and redundancy

The Results section includes substantial interpretive content that overlaps with the Discussion. Reducing repetition and moving interpretation to the Discussion would improve clarity and structure.

7. Language and readability

Although the manuscript has undergone language editing, minor grammatical issues and repetitive phrasing remain. Additional polishing would improve readability.

8. Figures and tables

Figure 1 provides limited information beyond what is already presented in the tables and may be redundant. The authors may wish to reconsider its inclusion.

9. Terminology consistency

Terminology related to under-five mortality should be used consistently throughout the manuscript to avoid ambiguity.

Overall Recommendation

Major revision

The study addresses an important public health question and uses a potentially valuable analytical approach. However, substantial revisions are required to improve conceptual clarity, statistical transparency, consistency of interpretation, and alignment between the study design and the conclusions.

**********

7. PLOS authors have the option to publish the peer review history of their article (what does this mean?). If published, this will include your full peer review and any attached files.

If you choose “no”, your identity will remain anonymous but your review may still be made public.

**Do you want your identity to be public for this peer review?**  For information about this choice, including consent withdrawal, please see our Privacy Policy.

Reviewer #1: **Yes:**  Bayan Abuhalimeh

Reviewer #2: No

**********

[NOTE: If reviewer comments were submitted as an attachment file, they will be attached to this email and accessible via the submission site. Please log into your account, locate the manuscript record, and check for the action link "View Attachments". If this link does not appear, there are no attachment files.]

To ensure your figures meet our technical requirements, please review our figure guidelines: https://journals.plos.org/plosone/s/figures

You may also use PLOS’s free figure tool, NAAS, to help you prepare publication quality figures: https://journals.plos.org/plosone/s/figures#loc-tools-for-figure-preparation.

NAAS will assess whether your figures meet our technical requirements by comparing each figure against our figure specifications.

Attachment Submitted filename: plos revision.docx

# Author comment: Author response to Decision Letter 2

**DOI:** 10.1371/journal.pone.0355621.r004

13 Mar 2026

Date: March 6, 2026

To: Editor-in-Chief

PLOS ONE

Dear Editor-in-Chief,

Re: Manuscript Submission - [Exploring the association between home delivery and under-five mortality in Ethiopia: A bivariate multilevel mixed-effects analysis], with manuscript ID: PONE-D-25-42341R1.

We would like to express our gratitude to the reviewers and editor for their valuable comments and suggestions on our manuscript entitled “Exploring the association between home delivery and under-five mortality in Ethiopia: A bivariate multilevel mixed-effects analysis”. We appreciate the time and effort invested in reviewing our work. In response to their comments, we have made significant revisions to address each point raised. Below, we provide a point-by-point response to the reviewers' comments. Our responses are prefaced by “Author Response” and are shown in blue to distinguish them from the reviewers’ comments.

We believe that these revisions have significantly strengthened our manuscript and have addressed all the concerns raised by the reviewers. We hope that our revised work meets the standards of PLOS ONE and would be suitable for publication. We appreciate your consideration of our revised manuscript.

Thank you once again for your time and attention to our submission.

Sincerely,

Wondaya Fenta Zewdia &

Maru Zewdu Kassie,

Corresponding Authors

In response to comments from Reviewer # 2

Reviewer #2: Summary

This manuscript investigates the association between home delivery and under-five mortality in Ethiopia using data from the 2019 Ethiopian Mini Demographic and Health Survey. The topic is highly relevant to maternal and child health in low- and middle-income countries, and the use of nationally representative data is a clear strength. The application of a bivariate multilevel mixed-effects model to jointly analyze two correlated outcomes is methodologically interesting and potentially valuable.

However, several major issues require careful attention before the manuscript can be considered for publication. These relate mainly to causal interpretation, outcome definition, statistical clarity, internal consistency of results, and overinterpretation of findings. Addressing these concerns will substantially improve the scientific rigor and clarity of the manuscript.

Author’s Response: We sincerely thank the reviewer for the insightful and constructive comments. We have carefully considered all suggestions and have thoroughly revised and refined the manuscript to improve its scientific rigor, clarity, and consistency.

Major Comments

1. Causal interpretation and language: The manuscript uses causal language despite employing a cross-sectional design. Such analyses can identify associations but cannot establish causality or temporal order, especially for under-five mortality. The Results, Discussion, and Conclusions should consistently use associative language, describing home delivery as associated with higher odds of under-five mortality rather than as a causal determinant.

Author response: We thank the reviewer for this important comment. We have addressed this concern in the revised manuscript by removing causal language and consistently using associative terminology throughout the Results, Discussion, and Conclusions, in line with the cross-sectional study design.

2. Definition and interpretation of under-five mortality: Under-five mortality is defined as a binary indicator of whether a woman experienced at least one child death before age five, rather than the standard rate per 1,000 live births. Reported percentages represent proportions of mothers reporting child loss (unit of analysis: mother), not mortality rates. This distinction should be clearly stated in the Methods, Results, and Discussion sections.

Author response: We appreciate the reviewer’s important clarification regarding the definition and interpretation of under-five mortality. In the revised manuscript, we have explicitly defined the outcome variable (under five mortality) in the Methods section (Variables in the Study, page 5, lines 180-183). Under-five mortality is clearly operationalized as a mother-level binary variable indicating whether a woman experienced at least one live-born child death before age five. We also explicitly state that this measure does not represent the conventional under-five mortality rate (deaths per 1,000 live births), but rather the proportion of mothers who experienced at least one under-five child death. Furthermore, we have ensured consistent wording throughout the Methods, Results, and Discussion sections to clarify that the unit of analysis is the mother and that all reported percentages refer to proportions of mothers, not child mortality rates.

3. Statistical modeling clarity and transparency: The statistical methods section contains extensive mathematical notation, but the conceptual explanation of the bivariate multilevel mixed-effects model is limited.

Author response: We have substantially revised the Statistical Analysis section to provide a clearer conceptual explanation of the bivariate multilevel mixed-effects model.

4. Internal consistency and interpretation of results: The manuscript shows inconsistencies, such as unusually small chi-square statistics, counterintuitive antenatal care findings, and misinterpretation of model intercepts. Statistical outputs and interpretations should be reviewed for consistency and aligned with proper statistical and epidemiological meaning.

Author response: We have carefully reviewed all statistical outputs and revised the manuscript to ensure internal consistency. Counterintuitive findings related to antenatal care have been clarified, and the interpretation of model intercepts has been corrected to reflect their proper statistical meaning.

5. Magnitude and stability of regional effects: Some regional adjusted odds ratios are extremely large, with values exceeding twenty in certain regions. These estimates raise concerns about sparse data, model instability, or over-fitting. The manuscript does not provide sufficient information on regional sample sizes or event counts to assess the robustness of these findings. The authors should report sample sizes by region, discuss the possibility of sparse-data bias, and interpret large regional estimates with appropriate caution.

Author response: We appreciate the reviewer’s important comment regarding the magnitude and stability of regional effects. The sample sizes and corresponding event distributions by region are provided in Table 3 (Appendix section, page 22-23, lines 607-609), titled “Distribution and Association of Covariates with Home Delivery and Under-Five Mortality.” We have now explicitly referenced this table in the main text to improve transparency regarding regional sample sizes and event counts. We have corrected this in the revised manuscript and acknowledge the substantial regional differences across Ethiopia. Several factors may contribute to this variability, including cultural differences, infrastructure disparities, instability, and religious diversity. In addition, we now discuss the potential for sparse-data bias in regions with relatively small sample sizes (e.g., Gambela, Harari, and Dire Dawa), and we interpret large adjusted odds ratios with appropriate caution, acknowledging the possibility of model instability due to limited event counts in some areas.

Minor Comments

6. Organization and redundancy: The Results section includes substantial interpretive content that overlaps with the Discussion. Reducing repetition and moving interpretation to the Discussion would improve clarity and structure.

Author response: We have restructured the manuscript to improve clarity and organization. The Results section now presents only objective statistical findings, while all interpretation and contextualization have been moved exclusively to the Discussion section. In addition, redundant content has been carefully removed to enhance conciseness and readability.

7. Language and readability: Although the manuscript has undergone language editing, minor grammatical issues and repetitive phrasing remain. Additional polishing would improve readability.

Author response: We have revised the manuscript and conducted additional careful language editing to correct minor grammatical issues and eliminate repetitive phrasing, thereby improving overall clarity and readability.

8. Figures and tables: Figure 1 provides limited information beyond what is already presented in the tables and may be redundant. The authors may wish to reconsider its inclusion.

Author response: Thank you for this thoughtful suggestion. We respectfully believe that Figure 1 is important for the manuscript, as it visually presents the distribution and association between the two outcome variables in a clear and intuitive manner. While related information appears in the tables, the figure enhances understanding by providing a graphical summary of the relationship. Therefore, we have retained Figure 1 in the revised manuscript.

9. Terminology consistency: Terminology related to under-five mortality should be used consistently throughout the manuscript to avoid ambiguity.

Author response: Thank you for the comment. We have standardized the terminology for under-five mortality throughout the manuscript to ensure clarity and consistency.

Reviewer Comment - Overall Recommendation: Major revision

Comment: The study addresses an important public health question and uses a potentially valuable analytical approach. However, substantial revisions are required to improve conceptual clarity, statistical transparency, consistency of interpretation, and alignment between the study design and the conclusions.

Author response: We appreciate the reviewer’s constructive feedback. We have carefully revised the manuscript to enhance conceptual clarity, improve statistical transparency, ensure consistent interpretation, and better align the study design with the conclusions.

Author response for the attached comments

Reviewer Comment: This manuscript addresses an important public health issue in Ethiopia and applies an interesting bivariate multilevel mixed-effects approach. However, significant methodological and reporting gaps remain.

Author Response: We sincerely thank the reviewer for recognizing the importance of our study and for the constructive comments. We have carefully addressed each point.

1. Could the authors clarify the sampling procedure, including the total number of participants invited, the response rate, recruitment method, inclusion/exclusion criteria, and how non-responses or incomplete cases were handled?

Author response: We have addressed the reviewer’s comment regarding the sampling procedure in the revised manuscript (page 5, lines 110–120).

2. Would the authors provide a flow diagram (as recommended by STROBE) showing how the final sample of 5,517 women was obtained?

Author response: We have incorporated a flow diagram showing the selection of the final sample of 5,517 women in the revised manuscript (page 6, lines 132–139).

3. How were missing values handled in the analysis?, Could the authors provide a table showing the proportion of missing data for each variable?

Author response: Thank you for your comment. Missing values were handled using the multiple imputation method. Regarding the proportion of missing data for each variable, only variables such as antenatal care, media exposure, and maternal education had small amounts of missing values. Since the proportions were very low, they were handled using multiple imputation. A description of this procedure has been added to the revised manuscript.

4. Did the authors apply DHS sampling weights in the analysis?, If not, could they explain why, and if so, could they clarify exactly how the weights were incorporated into the multilevel model?

Author response: Yes, the data for this study were obtained from the 2019 Ethiopian Mini Demographic and Health Survey (EMDHS). Individual sample weights (v005/1,000,000) were applied in all analyses to account for over- and under-sampling, and the weights were incorporated into the multilevel model accordingly.

5. Could the authors complete and submit a STROBE checklist to ensure compliance with reporting standards for observational studies?

Author response: We have completed the STROBE checklist and submitted it alongside the revised manuscript to ensure full compliance with reporting standards for observational studies.

Attachment Submitted filename: Response to Reviewers.pdf

# Peer review: Decision Letter 2

**DOI:** 10.1371/journal.pone.0355621.r005

**Authors:** Sajjad Haider Bhatti

26 May 2026

PONE-D-25-42341R2

Exploring the association between home delivery and under-five mortality in Ethiopia: A bivariate multilevel mixed-effects analysis

PLOS One

Dear Dr. Kassie,

Thank you for submitting your manuscript to PLOS ONE. After careful consideration, we feel that it has merit but does not fully meet PLOS ONE’s publication criteria as it currently stands. Therefore, we invite you to submit a revised version of the manuscript that addresses the points raised during the review process.

Please submit your revised manuscript by Jul 10 2026 11:59PM. If you will need more time than this to complete your revisions, please reply to this message or contact the journal office at plosone@plos.org. When you're ready to submit your revision, log on to https://www.editorialmanager.com/pone/ and select the 'Submissions Needing Revision' folder to locate your manuscript file.

Please include the following items when submitting your revised manuscript:

- A letter that responds to each point raised by the academic editor and reviewer(s). You should upload this letter as a separate file labeled 'Response to Reviewers'.
- A marked-up copy of your manuscript that highlights changes made to the original version. You should upload this as a separate file labeled 'Revised Manuscript with Track Changes'.
- An unmarked version of your revised paper without tracked changes. You should upload this as a separate file labeled 'Manuscript'.

If you would like to make changes to your financial disclosure, please include your updated statement in your cover letter. Guidelines for resubmitting your figure files are available below the reviewer comments at the end of this letter.

If applicable, we recommend that you deposit your laboratory protocols in protocols.io to enhance the reproducibility of your results. Protocols.io assigns your protocol its own identifier (DOI) so that it can be cited independently in the future. For instructions see: https://journals.plos.org/plosone/s/submission-guidelines#loc-laboratory-protocols. Additionally, PLOS ONE offers an option for publishing peer-reviewed Lab Protocol articles, which describe protocols hosted on protocols.io. Read more information on sharing protocols at https://plos.org/protocols?utm_medium=editorial-email&utm_source=authorletters&utm_campaign=protocols.

As the corresponding author, your ORCID iD is verified in the submission system and will appear in the published article. PLOS supports the use of ORCID, and we encourage all coauthors to register for an ORCID iD and use it as well. Please encourage your coauthors to verify their ORCID iD within the submission system before final acceptance, as unverified ORCID iDs will not appear in the published article. *Only*  the individual author can complete the verification step; PLOS staff *cannot*  verify ORCID iDs on behalf of authors.

We look forward to receiving your revised manuscript.

Kind regards,

Sajjad Haider Bhatti, Ph.D.

Academic Editor

PLOS One

**Journal Requirements:**

1. If the reviewer comments include a recommendation to cite specific previously published works, please review and evaluate these publications to determine whether they are relevant and should be cited. There is no requirement to cite these works unless the editor has indicated otherwise.

2. Please review your reference list to ensure that it is complete and correct. If you have cited papers that have been retracted, please include the rationale for doing so in the manuscript text, or remove these references and replace them with relevant current references. Any changes to the reference list should be mentioned in the rebuttal letter that accompanies your revised manuscript. If you need to cite a retracted article, indicate the article’s retracted status in the References list and also include a citation and full reference for the retraction notice.

**Additional Editor Comments:**

Dear Authors, I invite to revise your mansucript in light of the comments/concerns by reviewers.

[Note: HTML markup is below. Please do not edit.]

Reviewers' comments:

Reviewer's Responses to Questions

**Comments to the Author**

1. If the authors have adequately addressed your comments raised in a previous round of review and you feel that this manuscript is now acceptable for publication, you may indicate that here to bypass the “Comments to the Author” section, enter your conflict of interest statement in the “Confidential to Editor” section, and submit your "Accept" recommendation.

Reviewer #2: (No Response)

Reviewer #3: All comments have been addressed

**********

2. Is the manuscript technically sound, and do the data support the conclusions?

The manuscript must describe a technically sound piece of scientific research with data that supports the conclusions. Experiments must have been conducted rigorously, with appropriate controls, replication, and sample sizes. The conclusions must be drawn appropriately based on the data presented.

Reviewer #2: No

Reviewer #3: Partly

**********

3. Has the statistical analysis been performed appropriately and rigorously?

Reviewer #2: No

Reviewer #3: I Don't Know

**********

4. Have the authors made all data underlying the findings in their manuscript fully available?

The PLOS Data policy requires authors to make all data underlying the findings described in their manuscript fully available without restriction, with rare exception (please refer to the Data Availability Statement in the manuscript PDF file). The data should be provided as part of the manuscript or its supporting information, or deposited to a public repository. For example, in addition to summary statistics, the data points behind means, medians and variance measures should be available. If there are restrictions on publicly sharing data—e.g. participant privacy or use of data from a third party—those must be specified.

Reviewer #2: Yes

Reviewer #3: Yes

**********

5. Is the manuscript presented in an intelligible fashion and written in standard English?

PLOS ONE does not copyedit accepted manuscripts, so the language in submitted articles must be clear, correct, and unambiguous. Any typographical or grammatical errors should be corrected at revision, so please note any specific errors here.

Reviewer #2: Yes

Reviewer #3: Yes

**********

6. Review Comments to the Author

Please use the space provided to explain your answers to the questions above. You may also include additional comments for the author, including concerns about dual publication, research ethics, or publication ethics. (Please upload your review as an attachment if it exceeds 20,000 characters)

Reviewer #2: Thank you to the authors for the revisions. While several aspects of the manuscript have improved, important concerns remain insufficiently addressed, particularly regarding causal interpretation, statistical validity, and consistency of interpretation. These issues need further revision before the manuscript can be considered for publication.

Major Comments

1. Persistent causal language and overinterpretation

Despite revisions, the manuscript continues to use causal language inconsistent with a cross-sectional design “implying causality”. Statements such as:

• “children born at home are more likely to die”

• “protective effect of institutional delivery”

• “43% of deaths were attributed to home delivery”

imply causality or attribution, which cannot be supported by the study design. The manuscript must consistently use associative language and avoid any implication of causation or attribution.

2. Inconsistency between outcome definition and interpretation

The outcome is defined as a mother-level binary variable (experience of at least one under-five death). However, results and interpretations are frequently presented at the child level (e.g., “children born at home…”). This inconsistency is conceptually incorrect and must be resolved throughout the manuscript.

3. Statistical inconsistency in chi-square results

The reported chi-square statistic (χ² = 3.83) with p < 0.0001 appears implausible and suggests either a reporting or calculation error. All statistical results should be carefully verified and corrected.

4. Continued misinterpretation of model intercepts

The intercept term is still interpreted substantively. Intercepts represent baseline log-odds under reference conditions and should not be given epidemiological interpretation.

5. Limited improvement in statistical modeling clarity

While the Methods section includes additional explanation, the model remains difficult to interpret. In particular:

• the practical meaning of the association parameter is still unclear

• the “loop rule” is referenced without sufficient explanation

A more intuitive and applied explanation is needed.

6. Large regional effects remain insufficiently justified

Although sample sizes are now provided, extremely large adjusted odds ratios (e.g., >20) remain concerning. The manuscript should:

• discuss potential model instability more explicitly

• consider sensitivity analyses or model diagnostics

• interpret these estimates with greater caution

Minor Comments

• Some improvements in structure are noted, but minor language issues remain.

• Terminology should be consistently aligned with the unit of analysis (mother vs child).

• Thank you for incorporating the participant flow. It can be cosmetically improved by aligning the arrows. It should also show the ones that were excluded to be complete.

Reviewer #3: The manuscript investigates an important public health issue using nationally representative Ethiopian DHS data and applies a useful bivariate multilevel modeling framework. The revised version has improved in terms of organization, terminology consistency, and reduction of causal language. The study has potential scientific value and contributes to understanding the association between home delivery and maternal experience of under-five mortality in Ethiopia. Some concerns still remain unaddressed.

1. Some interpretations continue to imply causality despite the cross-sectional design, particularly in the Discussion and Conclusion sections.

2. There are inconsistencies in the interpretation of ANC findings and the operational definition of under-five mortality, where mother-level outcomes are occasionally discussed as child-level mortality rates.

3. Regional odds ratios appear extremely large that suggest possible sparse-data bias or model instability, and these findings require more cautious interpretation.

4. The statistical methods section still needs simplification and clearer explanation of the bivariate multilevel framework.

5. Minor grammatical issues and repetitive statements are also present throughout the manuscript.

6. The study is relevant and methodologically interesting, but moderate revision is still required to improve statistical clarity, interpretation consistency, and overall readability before publication consideration.

**********

7. PLOS authors have the option to publish the peer review history of their article (what does this mean?). If published, this will include your full peer review and any attached files.

If you choose “no”, your identity will remain anonymous but your review may still be made public.

**Do you want your identity to be public for this peer review?**  For information about this choice, including consent withdrawal, please see our Privacy Policy.

Reviewer #2: No

Reviewer #3: No

**********

[NOTE: If reviewer comments were submitted as an attachment file, they will be attached to this email and accessible via the submission site. Please log into your account, locate the manuscript record, and check for the action link "View Attachments". If this link does not appear, there are no attachment files.]

To ensure your figures meet our technical requirements, please review our figure guidelines: https://journals.plos.org/plosone/s/figures

You may also use PLOS’s free figure tool, NAAS, to help you prepare publication quality figures: https://journals.plos.org/plosone/s/figures#loc-tools-for-figure-preparation.

NAAS will assess whether your figures meet our technical requirements by comparing each figure against our figure specifications.

# Author comment: Author response to Decision Letter 3

**DOI:** 10.1371/journal.pone.0355621.r006

10 Jul 2026

Date: July 10, 2026

To: Editor-in-Chief

PLOS ONE

Dear Editor-in-Chief,

Re: Manuscript Submission - [Exploring the association between home delivery and under-five mortality in Ethiopia: A bivariate multilevel mixed-effects analysis], with manuscript ID: PONE-D-25-42341R2.

We would like to express our gratitude to the reviewers and editor for their valuable comments and suggestions on our manuscript entitled “Exploring the association between home delivery and under-five mortality in Ethiopia: A bivariate multilevel mixed-effects analysis”. We appreciate the time and effort invested in reviewing our work. In response to their comments, we have made significant revisions to address each point raised. Below, we provide a point-by-point response to the reviewers' comments. Our responses are prefaced by “Author Response” and are shown in blue to distinguish them from the reviewers’ comments.

We believe that these revisions have significantly strengthened our manuscript and have addressed all the concerns raised by the reviewers. We hope that our revised work meets the standards of PLOS ONE and would be suitable for publication. We appreciate your consideration of our revised manuscript.

Thank you once again for your time and attention to our submission.

Sincerely,

Wondaya Fenta Zewdia &

Maru Zewdu Kassie,

Corresponding Authors

In response to comments from Reviewer # 2

General Comment

Reviewer Comment: Thank you to the authors for the revisions. While several aspects of the manuscript have improved, important concerns remain insufficiently addressed, particularly regarding causal interpretation, statistical validity, and consistency of interpretation. These issues need further revision before the manuscript can be considered for publication.

Author Response: We sincerely thank the reviewer for the positive assessment and constructive recommendations. Following these suggestions, we thoroughly revised the manuscript to improve the consistency of the outcome definition, eliminate causal language, clarify the statistical methodology, simplify the interpretation of the bivariate multilevel model, and improve the presentation of the results. All requested revisions have been incorporated throughout the manuscript.

Major Comments

1. Persistent causal language and over-interpretation: Despite revisions, the manuscript continues to use causal language inconsistent with a cross-sectional design “implying causality”. Statements such as:

• “children born at home are more likely to die”

• “protective effect of institutional delivery”

• “43% of deaths were attributed to home delivery”

imply causality or attribution, which cannot be supported by the study design. The manuscript must consistently use associative language and avoid any implication of causation or attribution.

Author Response: We carefully reviewed the entire manuscript and removed all remaining causal or attributional language. Expressions such as “children born at home are more likely to die,” “protective effect of institutional delivery,” “reduces mortality,” and “43% of deaths were attributed to home delivery” have been removed or revised. Throughout the manuscript, we now consistently use association-based terminology such as “was associated with,” “had higher odds,” “was linked to,” and “may be associated with.” We also explicitly state in the Results, Discussion, Conclusion, and Strengths and Limitations sections that, because this study used a cross-sectional design, the findings represent statistical associations and should not be interpreted as causal relationships.

2. Inconsistency between outcome definition and interpretation: The outcome is defined as a mother-level binary variable (experience of at least one under-five death). However, results and interpretations are frequently presented at the child level (e.g., “children born at home…”). This inconsistency is conceptually incorrect and must be resolved throughout the manuscript.

Author Response: We carefully revised the manuscript to ensure that the interpretation is fully consistent with the outcome analyzed. The Methods section now clearly defines under-five mortality as a mother-level binary variable indicating whether a woman experienced at least one under-five child death. We subsequently revised the Abstract, Results, Discussion, Conclusion, and Strengths and Limitations sections to consistently interpret the findings at the mother level. Statements referring to child-level mortality were replaced with wording such as “maternal experience of under-five child death” or “mothers who reported at least one under-five child death,” thereby ensuring conceptual consistency throughout the manuscript.

3. Statistical inconsistency in chi-square results: The reported chi-square statistic (χ² = 3.83) with p < 0.0001 appears implausible and suggests either a reporting or calculation error. All statistical results should be carefully verified and corrected.

Author Response: We thank the reviewer for identifying this reporting error. The reported chi-square statistic (χ² = 3.83) was a typographical error. The correct value is χ² = 14.83, which is consistent with the reported p < 0.0001. We have verified all statistical results and corrected this value in the revised manuscript.

Changes made: Corrected the chi-square statistic from χ² = 3.83 to χ² = 14.83 after verifying the analysis.

4. Continued misinterpretation of model intercepts: The intercept term is still interpreted substantively. Intercepts represent baseline log-odds under reference conditions and should not be given epidemiological interpretation.

Author Response: We fully agree that the regression intercept does not have a meaningful epidemiological interpretation in this context. Accordingly, we removed the paragraph interpreting the intercept from the Results section. The revised Results now focus exclusively on the interpretation of the estimated regression coefficients and adjusted odds ratios for the explanatory variables.

5. Limited improvement in statistical modeling clarity: While the Methods section includes additional explanation, the model remains difficult to interpret. In particular:

• the practical meaning of the association parameter is still unclear

• the “loop rule” is referenced without sufficient explanation

A more intuitive and applied explanation is needed.

Author Response: We sincerely thank the reviewer for this valuable suggestion. In response, we substantially revised the Statistical Analysis section to improve the clarity and accessibility of the multilevel bivariate modeling approach. The explanation of the association parameter has been rewritten using simpler and more intuitive language. Specifically, we now explain that the association parameter, expressed as a log odds ratio, quantifies the adjusted relationship between home delivery and maternal experience of under-five child death after accounting for individual- and community-level characteristics and community-level clustering. We also clarify that an odds ratio greater than one indicates a positive association, an odds ratio equal to one indicates no association, and an odds ratio less than one indicates a negative association. In addition, we removed the reference to the “loop rule” because it was unnecessary for interpreting the model and could confuse readers. Finally, we expanded the rationale for using the bivariate multilevel mixed-effects logistic regression model and explained how it jointly models two correlated binary outcomes while accounting for the hierarchical structure of the data.

6. Large regional effects remain insufficiently justified: Although sample sizes are now provided, extremely large adjusted odds ratios (e.g., >20) remain concerning. The manuscript should:

• discuss potential model instability more explicitly

• consider sensitivity analyses or model diagnostics

• interpret these estimates with greater caution

Author Response: We appreciate the reviewer for this important comment. We have revised the Discussion to provide a more cautious interpretation of the regional estimates. Specifically, we now acknowledge that the very large adjusted odds ratios observed for some regions may partly reflect relatively small regional sample sizes, sparse data, or residual heterogeneity, which can result in unstable parameter estimates. We therefore advise readers to interpret these estimates cautiously. In addition, we re-examined the fitted model using standard diagnostic procedures and confirmed that the final model satisfied the convergence criteria and demonstrated an adequate overall fit based on model selection criteria. We have also noted this issue as a limitation of the study and recommend that future studies with larger regional samples further investigate these regional differences.

Minor Comments

Reviewer Comment: Some improvements in structure are noted, but minor language issues remain.

Author Response: We sincerely thank the reviewer for this suggestion. We carefully proofread the entire manuscript and revised numerous sentences to improve grammar, readability, and consistency. Redundant wording was removed, sentence structure was improved, and the overall flow of the manuscript was enhanced while preserving the original scientific content.

Reviewer Comment: Terminology should be consistently aligned with the unit of analysis (mother vs child).

Author Response: We thoroughly reviewed the manuscript and ensured that the terminology is consistently aligned with the mother-level outcome analyzed in this study. Child-level interpretations were revised throughout the manuscript to accurately reflect maternal experience of under-five child death.

Reviewer Comment: Thank you for incorporating the participant flow. It can be cosmetically improved by aligning the arrows. It should also show the ones that were excluded to be complete.

Author Response: The participant flow diagram has been revised to improve its visual presentation by aligning the arrows and enhancing the overall layout. We also updated the figure to explicitly indicate the number of participants excluded at each stage of the selection process, together with the reasons for exclusion, thereby providing a more complete and transparent description of participant selection.

Response to Reviewer #3

General Comment: The manuscript investigates an important public health issue using nationally representative Ethiopian DHS data and applies a useful bivariate multilevel modeling framework. The revised version has improved in terms of organization, terminology consistency, and reduction of causal language. The study has potential scientific value and contributes to understanding the association between home delivery and maternal experience of under-five mortality in Ethiopia. Some concerns still remain unaddressed.

Author Response: We sincerely thank the reviewer for the thoughtful evaluation of our manuscript and for recognizing its public health relevance, the use of nationally representative Ethiopian Mini DHS data, and the application of a bivariate multilevel modelling framework. We greatly appreciate the constructive comments and have carefully revised the manuscript to address all remaining concerns. Our detailed responses are presented below.

1. Some interpretations continue to imply causality despite the cross-sectional design, particularly in the Discussion and Conclusion sections.

Author Response: We sincerely thank the reviewer for this important observation. We carefully reviewed the entire manuscript and removed all remaining causal language. Expressions implying causality, such as “protective effect,” “reduces mortality,” “children born at home are more likely to die,” and “attributed to” have been replaced with appropriate association-based terminology, including “was associated with,” “had higher odds,” “was linked to,” and “may be associated with.” In addition, we explicitly state in the Discussion, Conclusion, and Strengths and Limitations sections that the cross-sectional study design identifies statistical associations but does not permit causal inference.

2. There are inconsistencies in the interpretation of ANC findings and the operational definition of under-five mortality, where mother-level outcomes are occasionally discussed as child-level mortality rates.

Author Response: We appreciate the reviewer for identifying this important issue. We carefully revised the interpretation of the antenatal care (ANC) findings to ensure that it is fully consistent with the model estimates and the reference category used in the analysis. Furthermore, we thoroughly reviewed the manuscript to ensure that the operational definition of under-five mortality is consistently presented as a mother-level binary outcome indicating whether a woman reported experiencing at least one under-five child death. All child-level interpretations were revised accordingly in the Abstract, Methods, Results, Discussion, and Conclusion to maintain consistency with the unit of analysis.

3. Regional odds ratios appear extremely large that suggest possible sparse-data bias or model instability, and these findings require more cautious interpretation.

Author Response: We sincerely thank the reviewer for this valuable suggestion. We have revised the Discussion to provide a more cautious interpretation of the regional estimates. Specifically, we now acknowledge that the large adjusted odds ratios observed for some regions may partly reflect relatively small sample sizes, sparse data, or residual heterogeneity, which can produce unstable parameter estimates. We therefore advise readers to interpret these findings cautiously. We also re-examined the fitted model using standard diagnostic procedures and confirmed that the final model achieved satisfactory convergence and model fit. This limitation has also been acknowledged in the manuscript, and we recommend that future studies with larger regional samples further investigate these regional differences.

4. The statistical methods section still needs simplification and clearer explanation of the bivariate multilevel framework.

Author Response: In response, we substantially revised the Statistical Analysis section to improve its clarity and accessibility. We expanded the rationale for using the bivariate multilevel mixed-effects logistic regression model and explained, in simpler language, that it simultaneously models two correlated binary outcomes while accounting for the hierarchical structure of the data. We also simplified the description of the association parameter by explaining its practical interpretation as the adjusted relationship between home delivery and maternal experience of under-five child death after accounting for individual- and community-level covariates and clustering. In addition, we removed the reference to the “loop rule” because it was unnecessary and could confuse readers.

5. Minor grammatical issues and repetitive statements are also present throughout the manuscript.

Author Response: We sincerely thank the reviewer for this helpful suggestion. We carefully proofread the entire manuscript and revised numerous sentences to improve grammar, readability, and consistency. Repetitive statements were removed, sentence structure was improved, and terminology was standardized throughout the manuscript while preserving the original scientific content.

6. The study is relevant and methodologically interesting, but moderate revision is still required to improve statistical clarity, interpretation consistency, and overall readability before publication consideration.

Author Response: We sincerely appreciate the reviewer's positive evaluation of the scientific relevance and methodological contribution of our study. Following the reviewer's recommendations, we comprehensively revised the manuscript to improve statistical clarity, strengthen consistency in the interpretation of the findings, simplify the presentation of the bivariate multilevel modelling framework, eliminate remaining causal language, and improve the overall readability of the manuscript. We believe that these revisions have substantially strengthened the manuscript and addressed all of the reviewer's concerns.

Thank You!!!

Attachment Submitted filename: Response_to_Reviewers_auresp_3.pdf

# Editor report: Decision Letter 3

**DOI:** 10.1371/journal.pone.0355621.r007

**Authors:** Sajjad Haider Bhatti

23 Jul 2026

Exploring the association between home delivery and under-five mortality in Ethiopia: A bivariate multilevel mixed-effects analysis

PONE-D-25-42341R3

Dear Dr. Kassie,

We’re pleased to inform you that your manuscript has been judged scientifically suitable for publication and will be formally accepted for publication once it meets all outstanding technical requirements.

Within one week, you’ll receive an e-mail detailing the required amendments. When these have been addressed, you’ll receive a formal acceptance letter and your manuscript will be scheduled for publication.

An invoice will be generated when your article is formally accepted. Please note, if your institution has a publishing partnership with PLOS and your article meets the relevant criteria, all or part of your publication costs will be covered. Please make sure your user information is up-to-date by logging into Editorial Manager at Editorial Manager® and clicking the ‘Update My Information' link at the top of the page. For questions related to billing, please contact billing support.

If your institution or institutions have a press office, please notify them about your upcoming paper to help maximize its impact. If they’ll be preparing press materials, please inform our press team as soon as possible -- no later than 48 hours after receiving the formal acceptance. Your manuscript will remain under strict press embargo until 2 pm Eastern Time on the date of publication. For more information, please contact onepress@plos.org.

Kind regards,

Sajjad Haider Bhatti, Ph.D.

Academic Editor

PLOS One

Additional Editor Comments (optional):

Reviewers' comments:

# Editor report: Acceptance letter

**DOI:** 10.1371/journal.pone.0355621.r008

**Authors:** Sajjad Haider Bhatti

PONE-D-25-42341R3

PLOS One

Dear Dr. Kassie,

I'm pleased to inform you that your manuscript has been deemed suitable for publication in PLOS One. Congratulations! Your manuscript is now being handed over to our production team.

At this stage, our production department will prepare your paper for publication. This includes ensuring the following:

* All references, tables, and figures are properly cited

* All relevant supporting information is included in the manuscript submission,

* There are no issues that prevent the paper from being properly typeset

You will receive further instructions from the production team, including instructions on how to review your proof when it is ready. Please keep in mind that we are working through a large volume of accepted articles, so please give us a few days to review your paper and let you know the next and final steps.

Lastly, if your institution or institutions have a press office, please let them know about your upcoming paper now to help maximize its impact. If they'll be preparing press materials, please inform our press team within the next 48 hours. Your manuscript will remain under strict press embargo until 2 pm Eastern Time on the date of publication. For more information, please contact onepress@plos.org.

You will receive an invoice from PLOS for your publication fee after your manuscript has reached the completed accept phase. If you receive an email requesting payment before acceptance or for any other service, this may be a phishing scheme. Learn how to identify phishing emails and protect your accounts at https://explore.plos.org/phishing.

If we can help with anything else, please email us at customercare@plos.org.

Thank you for submitting your work to PLOS ONE and supporting open access.

Kind regards,

PLOS ONE Editorial Office Staff

on behalf of

Dr. Sajjad Haider Bhatti

Academic Editor

PLOS One
